# Comparative transcriptomic analysis highlights contrasting levels of resistance of *Vitis vinifera* and *Vitis amurensis* to *Botrytis cinerea*

**DOI:** 10.1038/s41438-021-00537-8

**Published:** 2021-05-01

**Authors:** Ran Wan, Chunlei Guo, Xiaoqing Hou, Yanxun Zhu, Min Gao, Xiaoyan Hu, Songlin Zhang, Chen Jiao, Rongrong Guo, Zhi Li, Xiping Wang

**Affiliations:** 1 State Key Laboratory of Crop Stress Biology in Arid Areas, College of Horticulture, Northwest A&F University, 712100 Yangling, Xianyang, Shaanxi China; 2 College of Horticulture, Henan Agricultural University, 450002 Zhengzhou, Henan China; 3 College of Horticulture Science and Technology, Hebei Normal University of Science and Technology, 066004 Qinhuangdao, Hebei China; 4 Key Laboratory of Horticultural Plant Biology and Germplasm Innovation in Northwest China, Ministry of Agriculture, Northwest A&F University, 712100 Yangling, Xianyang, Shaanxi China; 5 Boyce Thompson Institute for Plant Research, Cornell University, Ithaca, NY 14853 USA; 6 Grape and Wine Research Institute, Guangxi Academy of Agricultural Sciences, 53000 Nanning, Guangxi China

**Keywords:** Biotic, Transcriptomics

## Abstract

*Botrytis cinerea* is a major grapevine (*Vitis* spp.) pathogen, but some genotypes differ in their degree of resistance. For example, the *Vitis vinifera* cultivar Red Globe (RG) is highly susceptible, but *V. amurensis* Rupr Shuangyou (SY) is highly resistant. Here, we used RNA sequencing analysis to characterize the transcriptome responses of these two genotypes to *B. cinerea* inoculation at an early infection stage. Approximately a quarter of the genes in RG presented significant changes in transcript levels during infection, the number of which was greater than that in the SY leaves. The genes differentially expressed between infected leaves of SY and RG included those associated with cell surface structure, oxidation, cell death and C/N metabolism. We found evidence that an imbalance in the levels of reactive oxygen species (ROS) and redox homeostasis probably contributed to the susceptibility of RG to *B. cinerea*. SY leaves had strong antioxidant capacities and improved ROS homeostasis following infection. Regulatory network prediction suggested that WRKY and MYB transcription factors are associated with the abscisic acid pathway. Weighted gene correlation network analysis highlighted preinfection features of SY that might contribute to its increased resistance. Moreover, overexpression of *VaWRKY10* in *Arabidopsis thaliana* and *V. vinifera* Thompson Seedless enhanced resistance to *B. cinerea*. Collectively, our study provides a high-resolution view of the transcriptional changes of grapevine in response to *B. cinerea* infection and novel insights into the underlying resistance mechanisms.

## Introduction

The necrotrophic fungus *Botrytis cinerea* is the causal agent of gray mold disease in grape (*Vitis vinifera*), which causes major losses in quality, yield and economic value^1^. The primary contact of *B. cinerea* with its host takes place at the cell surface, where plant responses are initiated. Factors that affect host resistance include the capacity to maintain cell wall integrity and the generation and accumulation of reactive oxygen species (ROS)^2^. Conversely, the pathogenicity of the fungus is associated with its tolerance to ROS and its ability to secrete cell wall-degrading enzymes and toxins that result in degradation and death of host tissue^3^. Maintenance of ROS homeostasis by the host is necessary for defense against *B. cinerea*, and an insufficient antioxidant system results in ROS-induced damage and cell death^4^. Activation of autophagy or apoptosis, which are two different types of programmed cell death (PCD) pathways, results in cell death and the appearance of necrotic plant tissue^5,6^. However, recent research has revealed differences between these two PCD pathways, resulting in enormous consequences for infection with necrotrophic fungi (such as *B. cinerea* or *Sclerotinia sclerotiorum*)^6^. It is suggested that if *B. cinerea* suppresses host autophagic PCD within 6–8 h after arrival on the leaf and then induces host apoptotic PCD (>16 hpi (hours post-inoculation)), host cell death and necrotic host tissue will occur^6^. On the other hand, leaves of different *Vitis* genotypes that are classified as resistant to *B. cinerea* have been reported to display increased antioxidant capacity and exhibit increased tolerance to oxidative stress caused by *B. cinerea*^7,8^.

Signal transduction networks controlled by various plant hormones are associated with host defense against *B. cinerea* infection. For example, the phytohormones ethylene (ETH) and jasmonic acid (JA) play important roles in host resistance to *B. cinerea*^9^, while abscisic acid (ABA) can suppress the defense response to *B. cinerea* by promoting signaling through the salicylic acid (SA) pathway and inhibiting JA signaling^10^. In *Arabidopsis thaliana*, the WRKY33 transcription factor has been reported to act as a key positive transcriptional regulator of defenses against *B. cinerea* strain 2100 (refs. ^11–13^). During *B. cinerea* infection, WRKY33 is activated by MPK proteins and regulates the expression of genes associated with metabolic responses and redox homeostasis; moreover, WRKY33 promotes JA- and ETH-mediated signaling pathways but suppresses the ABA signaling pathway to activate downstream defense responses^11–13^. Transcriptional reprogramming is thus an integral part of the host defense machinery, and members of some transcription factor families, such as the MYC, ERF, MYB, and NAC families, have also been shown to regulate different hormone signaling pathways and cell metabolism in response to *B. cinerea* challenge^14,15^.

Genome-scale studies, coupled with functional analyses, have great potential as a platform to elucidate the complexity of host defense mechanisms and can provide a foundation for breeding programs to enhance crop improvement and protection^16^. Several such studies in grape have revealed transcriptional changes and metabolic reprogramming that occur in response to *B. cinerea* infection^17–20^. For example, the expression of sets of genes related to regulatory systems such as hormone-mediated signaling and transcriptional regulation and biochemical pathways such as the phenylpropanoid pathway has been found to be upregulated in response to *B. cinerea* infection in grape tissues such as flowers, ripe berries and noble rotted berries (an atypical infection for botrytised wines) of *V. vinifera* cultivars, which are classified as susceptible to *B. cinerea*^17–20^. The expression of other gene sets related to biological processes, such as ROS responses, cell wall metabolism, and JA- and SA-associated pathways, has been observed to be either up- or downregulated in different tissues and *Vitis* varieties during *B. cinerea* infection^17–20^. Overall, these studies are based on the responses of susceptible grapes against *B. cinerea*, but limited information has been obtained about the expression and function of genes associated with resistant *Vitis* genotypes, such as *V. amurensis* Rupr and *V. adstricta*^7^.

Here, we used comparative transcriptome profiling to compare gene expression in the *V. amurensis* Shuangyou (SY) cultivar, which is highly resistant to *B. cinerea*, and the popular table grape *V. vinifera* cultivar Red Globe (RG), which is highly susceptible to the fungi^7^. We describe the transcriptome profiles of SY and RG at six stages of early interaction between the host and *B. cinerea* within 36 hpi. The results suggest that, compared with that of *V. vinifera*, the transcriptome of *V. amurensis* exhibits a relatively moderate change as part of its defense strategy, providing new insights into the resistance mechanisms of grapevine to *B. cinerea*.

## Results

### Shuangyou leaves but not Red Globe leaves effectively inhibited *B. cinerea* infection during early interaction stages

**Fig. 1:**
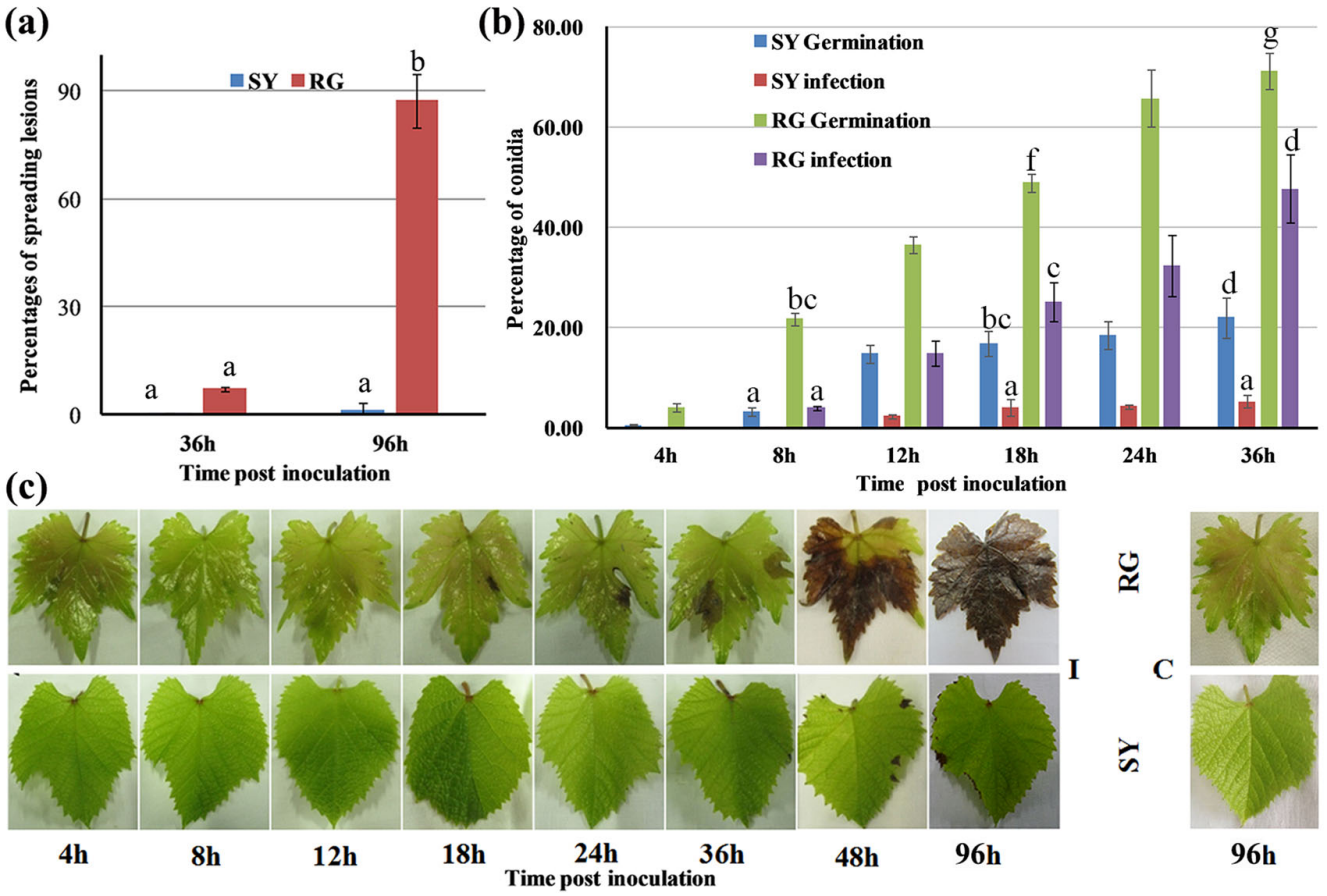
Disease development on RG and SY leaves infected by *Botrytis cinerea*. **a** Spreading lesion at 36 hpi (hours post-inoculation) and 96 hpi. **b** Percentage of germinated conidia (of the total number of conidia) and infection (of the total number of germinated conidia) at 4, 8, 12, 18, 24, and 36 hpi. At least 300 conidia were counted at each indicated time point. **c** RG and SY leaves at indicated time points. The means and standard errors were calculated from three biological replicates and 12 leaves of each replicate. The different lowercase letters represent significant differences at *P* ≤ 0.05 (Duncan’s test). RG *Vitis vinifera* cv. Red Globe, SY *V. amurensis* Shuangyou, I leaves inoculated with *Botrytis cinerea*, C control leaves inoculated with sterile water

We confirmed that *B. cinerea* initiated a successful infection on RG leaves but failed to colonize SY leaves before 36 hpi (Fig. 1). The RG leaves showed signs of necrosis at 96 hpi, whereas the SY leaves had few lesions (Fig. 1a). We verified this difference through an infection time course (Fig. 1b, c). On RG leaves inoculated with *B. cinerea*, conidial germination occurred at 4 hpi, and their numbers subsequently continued to increase, with appressoria penetrating the leaf surface followed by successful infection (defined as successful colonization of the fungus with infection peg formation on the leaves^7^) occurring from 8 hpi onward (Fig. 1b). Lesions were observed from 18 hpi and spread rapidly from 36 hpi on RG-infected leaves (Fig. 1c); more than half of the *B. cinerea* conidia had germinated at 18 hpi, and the infection rate reached 48% at 36 hpi (Fig. 1b and Supplemental Table 1A); in contrast, there were almost no lesions and much lower rates of germination and infection on the SY leaves at 36 hpi (Fig. 1b, c and Supplemental Table 1A).

### RNA-seq analysis

Using scanning electron microscopy (SEM), we monitored susceptibility interactions between *B. cinerea* and RG leaves at 4, 8, 12, 18, 24, and 36 hpi, encompassing the initial contact stage, conidial germination, surface penetration by appressoria, successful infection, primary lesion development and lesion expansion associated with hyphal extension and branching (Fig. 1 and Supplemental Fig. 1a). Comparatively, incompatible interactions between *B. cinerea* and SY leaves were observed during this period (Supplemental Fig. 1b). The initial contact stage on the SY leaves lasted 8 h, which was longer than that on the RG leaves, because the conidial germination rates on the SY leaves increased obviously from 8 hpi when the rates were much less than those on the RG leaves (Supplemental Fig. 1a, b). Surface penetration by appressoria and successful infections on SY leaves first appeared at 12 hpi, at least 4 h later, and then they increased significantly albeit slowly than those on the RG leaves did. In contrast to appressoria on the RG leaves, those on the SY leaves were always surrounded by sheaths, which seemed to peel away from leaf surfaces (Supplemental Fig. 1a, b). When hyphal extension and branching occurred and the lesion on the RG leaves expanded in size beginning at 18 hpi, infections on the SY leaves seemed stop due to almost no hyphal extension or branching with few lesions (Fig. 1 and Supplemental Fig. 1a, b). In total, the development of *B. cinerea* on the SY leaves was strongly blocked at these early interaction stages (Supplemental Fig. 1b).

We next performed RNA-seq (RNA sequencing) analysis of the RG and SY leaves over a time course encompassing the earliest interaction stages with *B. cinerea*. Samples were collected between 4 and 36 hpi. RNA-seq analyses of these samples using the Illumina HiSeq 2000/2500 platform (Illumina, Inc. USA) yielded a total of 622,303,085 mapped reads (average length = 100 bp) (Supplemental Table 1B). Of these, 0.67% mapped to the *B. cinerea* genome, and the percentage of *B. cinerea* reads gradually increased over the infection time course (Supplemental Table 1B).

A correlation analysis, the results of which are shown as a heat map, revealed distinct differences in gene expression between infected and control samples and between SY and RG samples (Supplemental Fig. 2a). The three replicates of all samples clustered together except two control RG samples at 8 and 12 hpi, two of whose replicates clustered together (Supplemental Fig. 2a). A significant correlation was also shown between the gene expression results obtained by the RNA-seq analysis and quantitative real-time qRT-PCR assays (Supplemental Fig. 2b). Principal component analysis (PCA) indicated that most of the variation in gene expression was a consequence of the infection process, as reflected by the relatively large distance in the data points derived from the control and infected samples. Moreover, there was a closer relation between the RG control and inoculation samples than between the SY samples at 4 and 8 hpi, indicating fewer transcriptome changes occurred during the initial interaction stages with *B. cinerea* in the RG leaves than in the SY leaves (Supplemental Fig. 2c, d).

A total of 7291 differentially expressed genes (DEGs) were identified through comparative transcriptome analysis of the RG and SY leaves during infection with *B. cinerea*. Of these, 6684 and 3227 DEGs were detected in the infected RG and SY leaves, respectively, representing 25% and 15% of all mapped genes based on the grape reference genome (Supplemental Fig. 3 and Supplemental Table 2A). The numbers of DEGs in the infected RG leaves was significantly greater than that in the infected SY leaves at early interaction stages of *B. cinerea*, with the exception of 8 hpi, the stage at which SY shows a higher proportion of DEGs, especially those whose expression is downregulated (Supplemental Fig. 3). This is consistent with more transcriptional reprogramming in the SY leaves than in the RG leaves at 8 hpi, when *B. cinerea* first penetrated the RG leaves but not the SY leaves (Fig. 1). GO term analysis of the DEGs was used to give a comparative overview of transcriptional changes and possible defense responses in infected grapevine, and 23 clusters of coexpression profiles of the DEGs were evaluated (Supplemental Fig. 4 and Supplemental Tables 2 and 3). DEGs involved in biological processes known to be associated with plant-pathogen interactions^2–48^ were selected for further studies (Supplemental Fig. 5 and Supplemental Table 4).

### The expression of genes associated with cell structure was downregulated much earlier in infected Shuangyou leaves than in infected Red Globe leaves

**Fig. 2:**
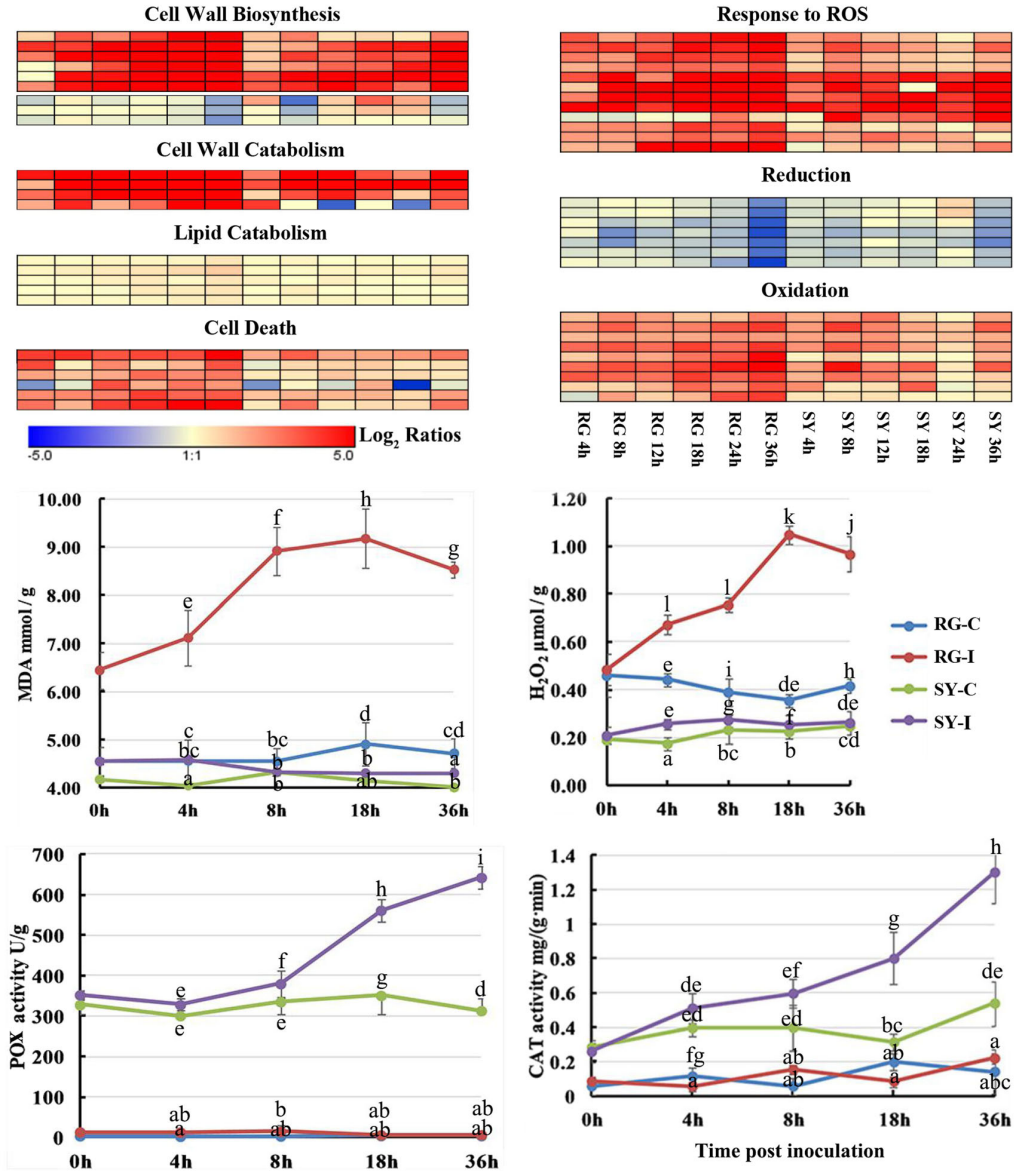
Heatmaps of differentially expressed genes (DEGs) and physiological changes associated with cell structure and reactive oxygen species. The heatmaps are based on data from Supplemental Table 4B and are associated with different GO term categories. The graphs show the levels of malondialdehyde (MDA) and H_2_O_2_ content as well as peroxidase (POX) and catalase (CAT) activities in RG and SY leaves at the indicated time points. The data represent the means of three experiments, and the error bars show the standard deviations. The different lowercase letters represent significant differences at *P* ≤ 0.05 (Duncan’s test). RG *Vitis vinifera* cv. Red Globe, SY *V. amurensis* Shuangyou, I leaves inoculated with *Botrytis cinerea*, C control, leaves inoculated with sterile water

The ‘cell wall metabolic process’ GO term was enriched at 8 hpi in the infected SY leaves but at 36 hpi in the infected RG leaves, including three genes whose expression was downregulated and that encoded cellulose synthase (CesA), a pivotal enzyme for cellulose biosythesis^21^. The expression of the *CesA* genes was downregulated at 8 hpi but upregulated from 18 hpi in the infected SY leaves, but their expression was not downregulated until 36 hpi in the infected RG leaves (Fig. 2 and Supplemental Table 4B). Other transcriptional responses to *B. cinerea* associated with cell wall metabolism were found to have increased in both infected RG and infected SY leaves. These responses included the expression of four laccase genes, three polygalacturonase (*PG*) genes and a pectinesterase (*PT*) gene (Fig. 2 and Supplemental Table 4B).

The ‘membrane lipid catabolic process’ GO term was associated only with the gene set whose expression was upregulated in the infected RG leaves at 36 hpi and not in the infected SY leaves during any of the early infection stages (Fig. 2 and Supplemental Table 4B). This set included a sphingosine phosphate lyase (*SPL*) gene, a phospholipase D-like protein (*PLD*) gene, a glycerophosphodiester phosphodiesterase (*GDPD*) gene, and a nonlysosomal glucosylceramidase (*GBA2*) gene. Moreover, the level of MDA (malondialdehyde) was much higher in the infected RG leaves than in the SY leaves during all infection stages (Fig. 2 and Supplemental Table 4).

### Infected Red Globe leaves and not Shuangyou leaves presented ROS accumulation and cell death

We observed substantial H_2_O_2_ accumulation in the infected RG leaves in addition to the upregulated expression of genes encoding respiratory burst oxidase homologs (NADPH oxidases), which can contribute to ROS generation^22^; however, this was not the case for infected SY leaves (Fig. 2 and Supplemental Table 4). The expression of several genes encoding peroxidase (POX) and glutathione S-transferase (GST), which are involved in the response to ROS, was strongly upregulated in both infected grapevine genotypes. Notably, however, the infected SY leaves showed earlier and more rapid increases in antioxidant activities, whereas antioxidant activities were not elevated at any time during the early interaction with *B. cinerea* in the RG leaves (Fig. 2 and Supplemental Table 4). Additionally, the expression of genes encoding proteins with a role in oxidation, such as FAD-binding monooxygenase, was more strongly upregulated, while the expression of genes encoding proteins with redox functions, such as malate dehydrogenase, was downregulated severalfold in the infected RG leaves compared to the infected SY leaves at 36 hpi (Fig. 2 and Supplemental Table 4). This suggests that SY leaves maintain redox homeostasis more effectively than RG leaves do during *B. cinerea* infection. Moreover, RG leaf cell death was associated with increasing lesion numbers during *B. cinerea* infection (Fig. 1c), and the expression of the gene set associated with the GO term ‘cell death’, as well as ‘programmed cell death’, was only upregulated in the infected RG leaves at 36 hpi. These genes included four disease resistance protein (*R*)-encoding genes, three genes encoding MLO-like proteins and a gene encoding an SPL that was also associated with the ‘membrane lipid catabolic process’ GO term (Fig. 2 and Supplemental Table 4).

### Expression of genes associated with the ABA pathway

Genes associated with the ABA pathway were differentially expressed in the infected RG and SY leaves, as indicated by the enrichment of the GO terms ‘ABA biosynthetic process’, ‘ABA catabolic process’ and ‘response to ABA’ (Supplemental Table 4). The expression of three cytochrome P450 genes associated with the ‘ABA catabolic process’ GO term, as well as two aldehyde oxidase (*AOX*) genes and two short-chain dehydrogenase (*SDR*) genes associated with the ‘ABA biosynthetic process’ GO term, was more highly upregulated in the infected RG leaves than in the infected SY leaves (Supplemental Table 4).

**Fig. 3:**
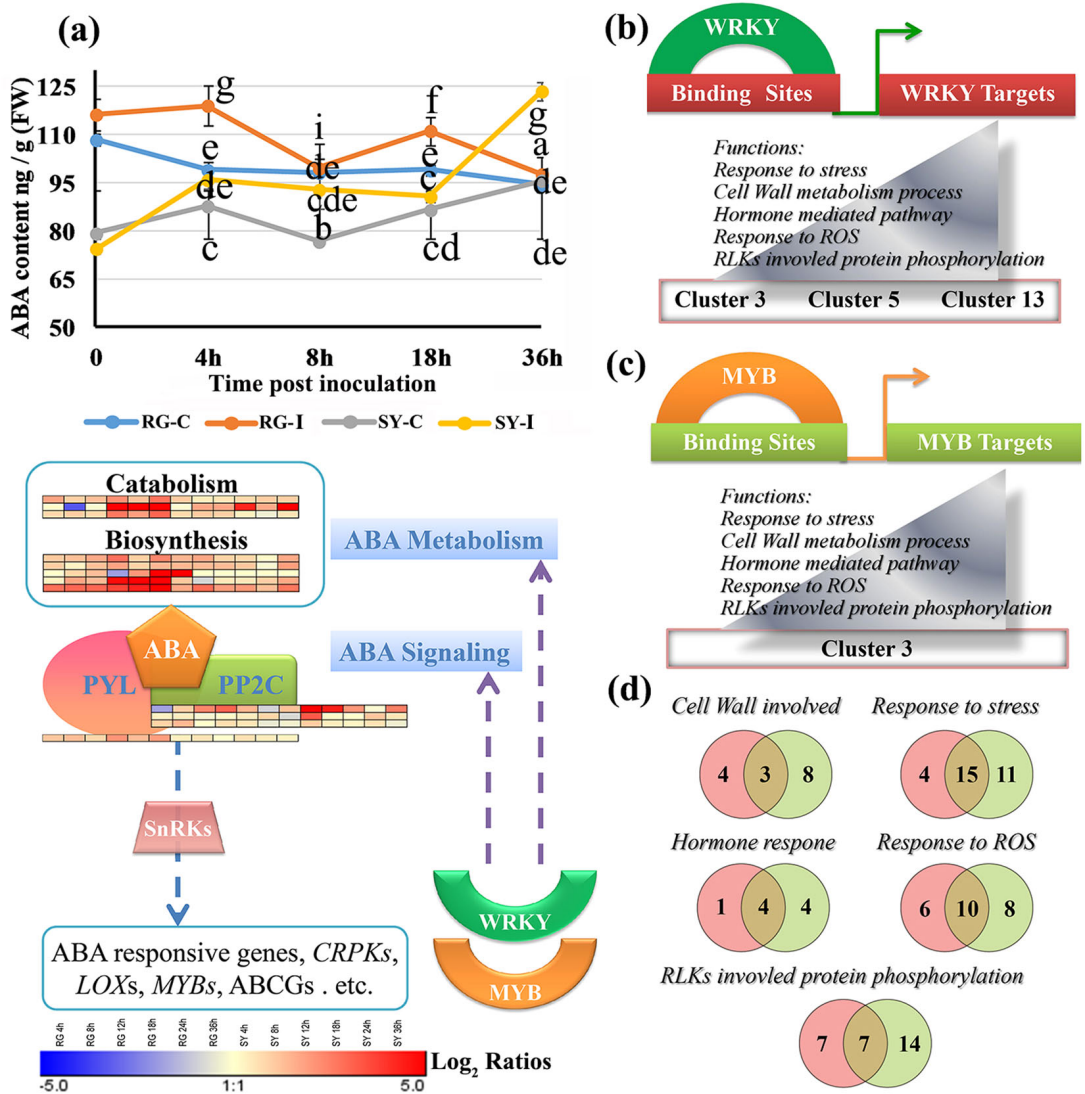
Predicted regulatory WKRY and MYB transcription factor modules related to abscisic acid (ABA) and reactive oxygen species (ROS) responses. **a** ABA levels in RG leaves and SY leaves at the indicated time points and differentially expressed genes (DEGs) associated with ABA metabolism and signaling. The data represent the means of three experiments, and the error bars represent the standard deviations. The different lowercase letters represent significant differences at *P* ≤ 0.05 (Duncan’s test). **b** and **c** Predicted regulatory WKRY and MYB TF modules with related functional categories of target genes. **d** Common genes whose expression is hypothetically regulated by WRKY (red) and MYB (green) TFs. The red and green circles indicate the number of predicted WRKY and MYB targets associated with the indicated biological functions. RG *Vitis vinifera* cv. Red Globe, SY *V. amurensis* Shuangyou, I leaves inoculated with *Botrytis cinerea*, C control, leaves inoculated with sterile water

We detected higher ABA levels in the infected RG leaves than in the infected SY leaves before 18 hpi (Fig. 3a). Notably, the expression of the ABA receptor-encoding gene *PYL4* was upregulated only in the infected RG leaves, while the expression of three genes encoding protein phosphatase 2c (PP2C) showed a greater and earlier induction in the infected SY leaves. Finally, two genes encoding cysteine-rich receptor-like protein kinases (*CRKs*), three *LOX* genes, and *MYB* or *MYB*-like genes were grouped in the set of genes whose expression was upregulated and that were associated with the ‘response to ABA’ GO term; the expression of these genes was upregulated more in the infected RG leaves than in the infected SY leaves (Fig. 3a and Supplemental Table 4).

### Predicted regulatory modules integrate transcription factor expression, regulatory motifs, and gene functions

The expression of many genes encoding transcription factors (*TF*s) in the *WRKY*, *MYB*, *ERF*, and *NAC* families was strongly upregulated in the RG and SY leaves in response to *B. cinerea* infection (Supplemental Table 5A). The expression of three grape *ORG2* genes encoding analogs of AtbHLH38/ORG2^28^ was downregulated at 24–36 hpi in the infected RG leaves, while the expression in the SY leaves was downregulated at 4–8 hpi and then upregulated at 12–36 hpi (Supplemental Table 5A).

We searched for *TF* genes that were present in the same gene expression cluster as genes that contain the TF target motifs in their promoters (Supplemental Fig. 4 and Supplemental Table 5) to predict transcriptional modules and their associated gene function categories^24^. The results revealed several regulatory modules containing WRKY and MYB genes, together with their predicted target genes, in clusters 3, 5, 11, and 13 (Supplemental Tables 6 and 7).

Potential regulatory modules linking *WRKY* and *MYB* genes with their target genes were also inferred from the following functions and pathways: ‘response to stress’, ‘cell wall metabolism process’, ‘response to ROS’, ‘RLKs involved in protein phosphorylation’ and ‘hormone-mediated pathways’ (Fig. 3b, c and Supplemental Table 7). Some target genes for the two classes of TFs overlapped (Fig. 3d), including peroxidase (*POX*) and laccase genes, consistent with crosstalk between defense responses (Fig. 3b, c and Supplemental Table 7). Potential targets were also genes related to stress responses, including genes encoding RLKs, ankyrin repeat-containing proteins (ANKs), R proteins and copine proteins (CPNs; Fig. 3b, c and Supplemental Table 7). In addition, two *CRPK* promoters were predicted to contain MYB-binding motifs, an *SDR* promoter was predicted to contain WRKY- and MYB-binding motifs, and a *PYL4* promoter was predicted to contain a WRKY-binding motif (Fig. 3a and Supplemental Table 7). These four genes were all involved in the ABA pathway in the grape leaf response to *B. cinerea* (Fig. 3a and Supplemental Table 4B).

### Infected Shuangyou and Red Globe leaves displayed different gene expression patterns associated with C/N metabolism

**Fig. 4:**
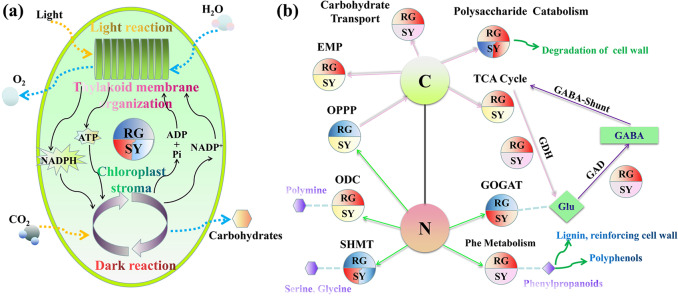
Differently expressed genes involved in C/N metabolism in RG and SY leaves infected by *B. cinerea*. **a** Differently expressed genes involved in photosynthesis. **b** Differently expressed genes involved in C/N metabolism. The blue semicircles show the genes whose expression was downregulated and that were associated with the indicated biological processes, while the red semicircles show the genes whose expression was upregulated. The pink and light blue semicircles show slight upregulated expression and slight downregulated expression of the corresponding genes, respectively. The yellow semicircles show genes with no significant change in expression. A semicircle composed of different colors represents a change between up- and downregulated expression during infection. RG *Vitis vinifera* cv. Red Globe, SY *V. amurensis* Shuangyou, EMP glycolysis, TCA tricarboxylic acid, OPPP oxidative pentose phosphate pathway, GABA γ-aminobutyrate, GDA glutamate decarboxylase, GDH glutamate dehydrogenase, SHMT serine hydroxymethyltransferase, ODC ornithine decarboxylase, GOGAT (GS1) glutamine-oxoglutarate aminotransferase (Glu synthase), Glu glutamate, Phe phenylalanine, C carbohydrate metabolism, N nitrogen metabolism

The expression of genes with functions related to photosynthesis light and dark reactions and thylakoid membrane organization was more downregulated at 36 hpi in the infected RG leaves than in the infected SY leaves, and these genes showed slightly increased expression in the infected SY leaves at 24 hpi (Fig. 4a and Supplemental Table 4). An important reduction in total chlorophyll was observed in both RG- and SY-infected samples during infection (except at 8 hpi), and the reduction between infected and control samples at each time point was less for SY than for RG except at 18 hpi (Supplemental Fig. 6a). The expression of genes encoding proteins associated with glycolysis (the EMP pathway) and the tricarboxylic acid (TCA) cycle, such as phosphofructokinase and citrate synthase, was upregulated more in the infected RG leaves than in the infected SY leaves (Fig. 4b and Supplemental Table 4B). Moreover, the expression levels of several carbohydrate transporter genes, for example, a hexose transporter gene and two facilitated glucose transporter member 8 genes, were higher in the infected RG leaves than in the infected SY leaves (Fig. 4b and Supplemental Table 4B), while the expression levels of genes encoding ribose-5-phosphate isomerase and fructose-1,6-bisphosphatase, which are associated with the oxidative pentose phosphate pathway (OPPP), were downregulated in the infected RG leaves but not in the infected SY leaves (Fig. 4b and Supplemental Table 4B).

The ‘amino acid metabolism’ GO term was detected more frequently for related genes whose expression was more strongly upregulated in the infected RG leaves than in the infected SY leaves (Fig. 4b and Supplemental Table 4). For example, the expression of genes encoding cinnamoyl-CoA reductase, chalcone synthase and dihydroflavonol-4-reductase, which are associated with the metabolism of phenylalanine, possibly for lignin and polyphenols^25^, was much higher in the infected RG leaves than in the infected SY leaves (Fig. 4b and Supplemental Table 4). In addition, total protein levels were higher in all the SY samples than in all the RG samples (Supplemental Fig. 6b). Taken together, these results suggest that, compared with SY leaves, RG leaves infected by *B. cinerea* might require more N nutrition and C skeletons but have a possibly lower supply.

Many genes associated with the metabolism of glutamate (Glu), which plays a central role in N metabolism through the glutamine (Gln) synthetase/Glu synthase cycle (GS/GOGAT cycle)^26^, showed different expression profiles in the two grapevine genotypes upon infection by *B. cinerea* (Fig. 4b and Supplemental Table 4). The expression of a nitrite reductase (*NR*) gene and two genes encoding Glu synthase (GS1, also named GOGAT, glutamine-oxoglutarate aminotransferase) was strongly induced only in the infected SY leaves, while the expression of a gene encoding glutamate decarboxylase (GAD) and several genes encoding glutamate dehydrogenase (GDH) was more highly induced in the infected RG leaves than in the infected SY leaves (Fig. 4b and Supplemental Table 4). In contrast, the expression of several serine hydroxymethyltransferase (*SHMT*) genes was downregulated at 36 hpi in the infected RG leaves but upregulated at 24 hpi in the infected SY leaves. A gene encoding ornithine decarboxylase (ODC) was also more highly expressed in the infected RG leaves than in the infected SY leaves, especially from 12 hpi onward (Fig. 4b and Supplemental Table 4).

### Weighted gene correlation network analysis provides insights into the resistance of Shuangyou leaves to *B. cinerea*

**Fig. 5:**
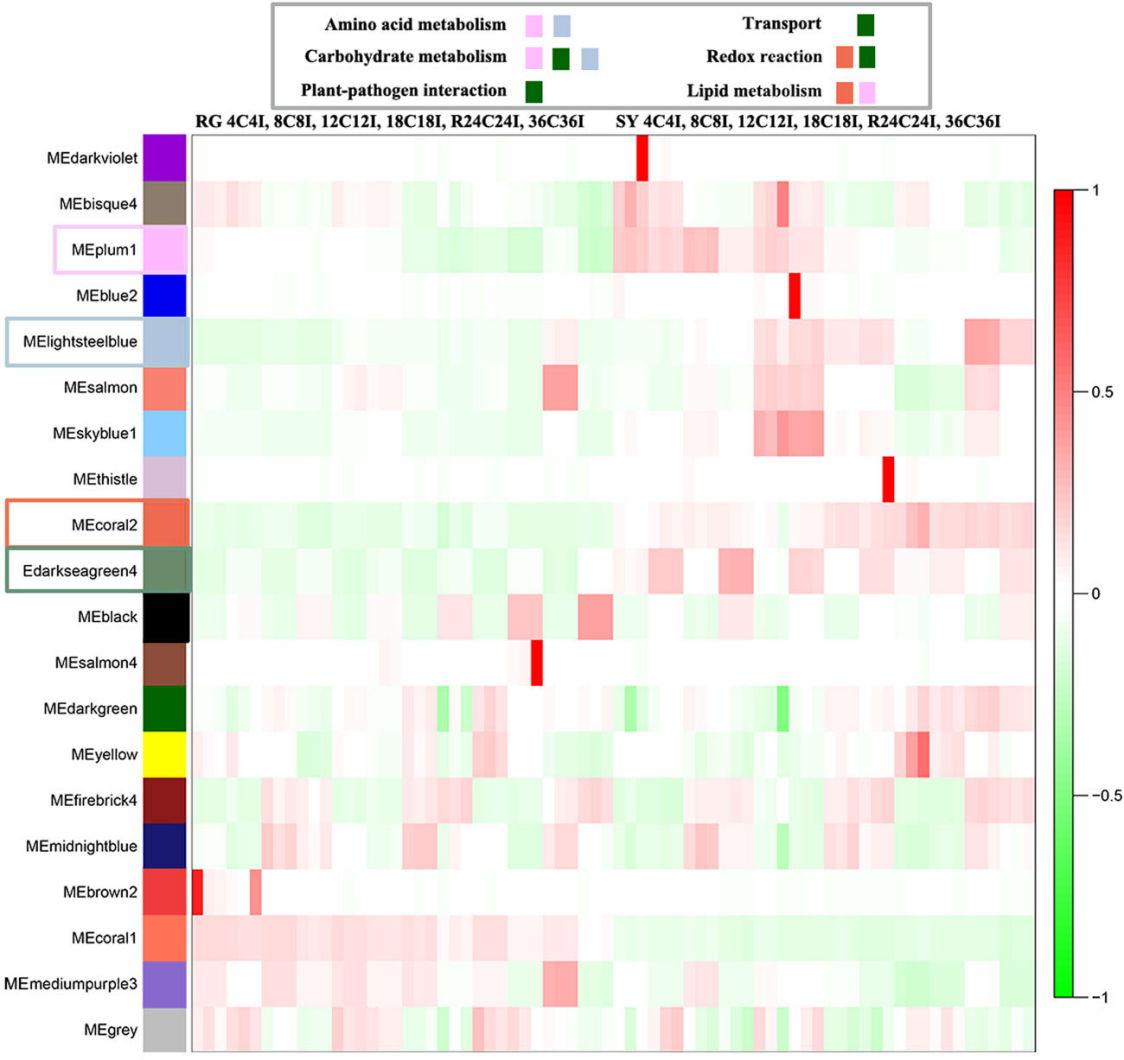
Expression modules constructed by weighted gene correlation network analysis (WGCNA) and associated biological processes of genes in the ‘black’, ‘coral2’, ‘plum1’, darkseagreen4’, and ‘lightsteelblue’ modules. RG *Vitis vinifera* cv. Red Globe, SY *V. amurensis* Shuangyou, I leaves inoculated with *B. cinerea*, C control leaves inoculated with sterile water

We next used Weighted gene correlation network analysis (WGCNA)^27^ to identify 20 modules of highly correlated genes based on the expression data (reads per kilobase of exon model per million mapped reads (RPKM), representing the expression level of a gene in a sample) from control and *B. cinerea*-inoculated leaves (Supplemental Figs. 7 and 8). The results highlighted genes in the ‘coral2’, ‘plum1’, ‘darkseagreen4’ and ‘lightsteelblue’ modules, whose RPKM values were greater in the control SY leaves than in the control RG leaves and greater in the infected SY leaves than in the infected RG leaves at each time point (Fig. 5). RPKM values of genes in control samples were defined as their basal expression levels. Enrichment analyses of GO terms based on the four modules revealed many genes that might be related to the resistance of SY leaves to *B. cinerea* with functions involving basic metabolism, transport, redox reactions and plant-pathogen interactions (Fig. 5; Supplemental Fig. 9; and Supplemental Table 8).

A number of genes involved in carbohydrate metabolism exhibited higher basal expression in the SY leaves than in the RG leaves (Supplemental Fig. 9 and Supplemental Table 8B). These included EMP (such as pyruvate kinase, phosphoenolpyruvate carboxylase, and fructokinase)-related genes and genes related to the TCA cycle (such as malate dehydrogenase, isocitrate dehydrogenase and mitochondrial pyruvate carrier 2) (Supplemental Fig. 9 and Supplemental Table 8B). Genes encoding thioesterase, ABC transporters and cycloartenol synthase (CAS) associated with the ‘lipid metabolism’ GO term exhibited higher basal expression in SY leaves than in RG leaves (Supplemental Fig. 9 and Supplemental Table 8B). This was also true for genes associated with *GS1*, *GST*, and *CAT* involved in Glu metabolism and redox reactions (Supplemental Fig. 9 and Supplemental Table 8B). In contrast, genes associated with the ‘plant-pathogen interaction’ category, including nine *ANK* (ankyrin repeat-containing protein) genes and three copine (*CPN*) genes, exhibited significantly higher basal expression in the SY samples than in the RG samples (Supplemental Fig. 9 and Supplemental Table 8B).

### Evaluation of *VaWRKY10* function involving *B. cinerea* resistance

**Fig. 6:**
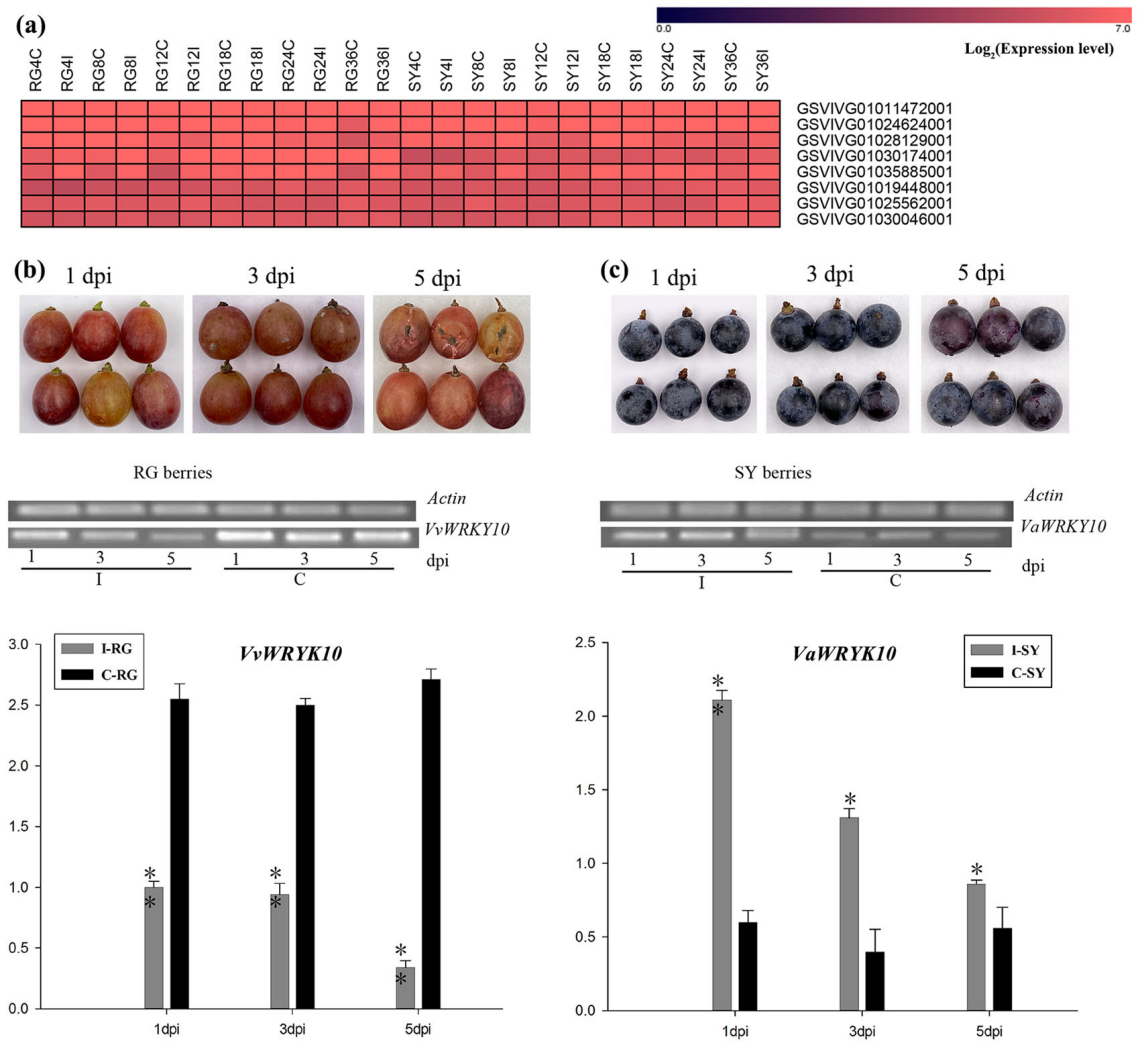
Grape *WRKY10* expression in RG and SY leaves and berries. **a** Heatmap of the expression of non-DEG *WRKY*s (data in Supplemental Table 8C). **b** *VvWRKY10* expression in RG berries and **c** *VaWRKY10* expression in SY berries at 1, 3, and 5 days post-inoculation (dpi) with *Botrytis cinerea*. The relative expression of *VvWRKY10* in the *B. cinerea*-inoculated RG sample at 1 dpi was set to 1.0 for normalization. One asterisk and two asterisks represent significant differences at *P* ≤ 0.05 and *P* ≤ 0.01 (*t*-test), respectively. RG *Vitis vinifera* cv. Red Globe, SY *V. amurensis* Shuangyou, I grape leaves and berries inoculated with *B. cinerea*, C control grape leaves and berries treated with sterile water

WGCNA further revealed that several *WRKY*s were not differentially expressed in response to *B. cinerea* infection but exhibited high basal expression in all the SY and RG control samples; these *WRKY*s were defined as non-DEG *WRKYs* (Fig. 6a and Supplemental Table 8C). Moreover, the basal expression levels of these non-DEG *WRKYs* in most of the SY and RG samples were much higher than those *WRKY*s that were differentially expressed in response to *B. cinerea* infection (Supplemental Table 8C). We therefore investigated whether these non-DEG *WRKY* genes are involved in resistance against *B. cinerea. WRKY10* (GSVIVG01035885001), a gene encoding one of these non-DEG WRKYs, was selected for further functional characterization, as a homolog of *AtWRKY18*, -*40*, and -*60* from *A. thaliana*^28^, which have been shown to play roles in *B. cinerea* resistance^29^. *VvWRKY10* and *VaWRKY10* were cloned from RG and SY leaves, respectively, and were found to have highly similar coding sequences, with only one base pair difference, which caused a single-amino acid difference (Supplemental Fig. 9).

Vine resistance to *B. cinerea* attack on the fruit matters very much to winegrowers. As such, the resistance of mature berries of RG and SY to *B. cinerea* was investigated by inoculation with conidial suspensions, and we observed clear gray mold disease on the surfaces of the RG berries but not on the SY berries at 5 dpi (days post-inoculation; Fig. 6b, c). In contrast to the expression patterns of *WRKY10* in the RG and SY leaves after *B. cinerea* infection, the expression of *VvWRKY10* was significantly downregulated in the RG berries, while that of *VaWRKY10* was significantly upregulated in the SY berries compared to the control (Fig. 6b, c). This suggests that grape *WRKY10* is involved in the response of grapevine to *B. cinerea*.

**Fig. 7:**
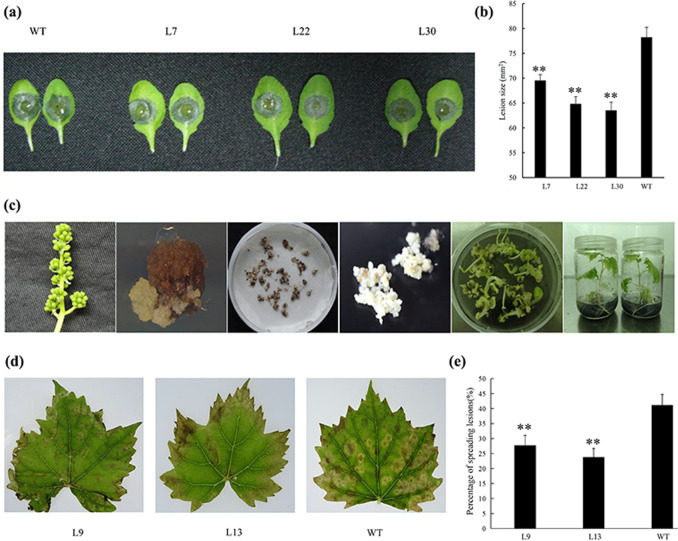
*VaWRKY10* transgenic *A. thaliana* and Thompson Seedless plants after infection by *B. cinerea*. **a** Images of *A. thaliana* leaves and **b** lesion sizes from *VaWRKY10* transgenic L7, L22 and L30 and Col-0 (WT) at 48 h post-inoculation (hpi) with *B. cinerea*. **c** Successive technological processes for *VaWRKY10* transgenic Thompson Seedless plants: inflorescences without blossoms; embryonic calli; proembryogenic masses; vigorous somatic embryos; introduction of *VaWRKY10* through *Agrobacterium*-mediated transformation; germination; and cultivation. **d** Images of *V. vinifera* Thompson Seedless leaves and **e** percentage of spreading lesions from *VaWRKY10* transgenic lines (L9, L13) and nontransgenic lines (WT) at 36 hpi with *B. cinerea*. The data represent the means of three replicates, with 15–18 leaves in each replicate. The error bars represent the standard deviations. The asterisks indicate statistical significance (**P < 0.01; *t*-test)

We generated *VaWRKY10* transgenic *A. thaliana* lines (Supplemental Fig. 11a), and after inoculating the plants with *B. cinerea*, we observed significantly larger lesions at 48 hpi on the leaves of wild-type (WT) *A. thaliana* Col-0 plants than on leaves of the *VaWRKY10* transgenic lines (L17, L22, L30; Fig. 7a, b). L30 displayed the highest relative expression level of *VaWRKY10*, followed by L17 and L22, during infection (Supplemental Fig. 11a), showing an inverse correlation with lesion size on their leaves (Fig. 7a, b and Supplemental Fig. 11a).

*V. vinifera* cv. Thompson Seedless *VaWRKY10* transgenic plants were generated through the *Agrobacterium*-mediated transformation method (Fig. 7c and Supplemental Fig. 11b). Thompson Seedless is susceptible to *B. cinerea*^8^, and compared with the nontransgenic leaves, the transgenic leaves (L9, L13) showed higher relative expression levels of *VaWRKY10* and significantly fewer lesions at 36 hpi (Fig. 7d, e and Supplemental Fig. 11b). L9 showed slightly lower relative expression levels of *VaWRKY10* but slightly more lesions than L13 did (Fig. 7d, e and Supplemental Fig. 11b). Taken together, our results suggested that the *VaWRKY10* gene can enhance the resistance of grapevine to *B. cinerea*.

## Discussion

Here, we performed a comparative transcriptome study using the *B. cinerea*-resistant *V. amurensis* SY cultivar. Major transcriptome differences during the early interaction stages of *B. cinerea* were apparent between the two grapevine genotypes with differing degrees of resistance, adding to the knowledge of the grapevine resistance mechanism towards *B. cinerea*.

### Early defense responses related to the cell wall might facilitate the resistance of Shuangyou leaves to *B. cinerea*

The cell wall plays a key role in plant defense both structurally and in terms of signaling capacity^15^. Upregulated expression of *PT*s and *PG*s can lead to impaired cell wall integrity and increased susceptibility to *B. cinerea*^2^. Since the expression of these genes was upregulated in both infected RG and infected SY leaves, the differential expression of *CesA* genes might be worthwhile to investigate (Fig. 2). It has been reported that the expression of *CeSA1* and *CeSA3* in *A. thaliana* is downregulated during *B. cinerea* infection, and the mutations in these genes decrease susceptibility to *B. cinerea* by JA/ETH defense signaling^15^. Moreover, the timing of *CeSA* repression may be crucial. In the *myb46 A. thaliana* mutant, which is more resistant than wild-type Col-0, *B. cinerea* induces transient and more pronounced downregulation of the expression of eight *CesA* genes that coincides with upregulated expression of the defensin *PDF1.2a* and the basal chitinase *PR-3* marker genes^21^. The downregulation of *CesA* genes that occurred earlier in the infected SY leaves than in the RG leaves (Fig. 2) might facilitate the resistance of SY leaves against *B. cinerea*.

### Red Globe leaves but not Shuangyou leaves showed evidence of ROS accumulation and cell death during *B. cinerea* infection

Higher expression of genes involved in H_2_O_2_ generation and more H_2_O_2_ accumulation occurred in the infected RG leaves than in the infected SY leaves (Fig. 2). It is well known that ROS accumulation can promote successful invasion of *B. cinerea* by the active HR (hypersensitive response) PCD pathway^5^. The expression of *R* genes from the gene set associated with the ‘cell death’ GO term was upregulated only in the infected RG leaves and not in the infected SY leaves; these genes often induce HR cell death^30^. Moreover, the grape *R* gene *VaRGA1* and the tomato *R* gene *SlNL33* function to increase susceptibility to *B. cinerea*^31,32^. Thus, we suggest that ROS accumulation and possibly cell death associated with *R* genes might be involved in the susceptibility of RG to *B. cinerea*.

*B. cinerea* infection can induce two kinds of host PCD: autophagy and apoptosis^6^. Generally, host autophagic PCD suppresses *B. cinerea* infection, while host apoptotic PCD promotes it^6^. Ceramide sphingolipids are crucial components for apoptotic PCD induction and affect the outcome of *B. cinerea* infection^6,33–35^. We also found a *GBA2* gene from the gene set associated with the ‘membrane lipid catabolic process’ GO term and an *SPL* gene categorized in the gene sets associated with the ‘cell death’ and ‘membrane lipid catabolic process’ GO terms, both of whose expression was upregulated only in the infected RG leaves and not in the infected SY leaves (Fig. 2). It has been reported that these two genes play important roles in apoptotic PCD^33–35^. GBA2 encodes an enzyme catalyzing the hydrolysis of glucosylceramide into glucose and ceramide^33^. In plant cells, any mutation resulting in increased levels of ceramide sphingolipids can cause cell growth arrest and apoptotic PCD^34^. SPL can enhance the generation of stress-induced ceramide and apoptosis in mammalian cells^35^. Therefore, these results should partly explain the susceptibility of RG leaves and the resistance of SY leaves against *B. cinerea* infection. However, their detailed roles and regulatory mechanisms in grapevine-*B. cinerea* interactions need further study.

*B. cinerea* infection can also induce host lipid peroxidation and ROS bursts^36^, which also occurred in the infected RG leaves. We detected that only the infected RG leaves and not the infected SY leaves displayed increased ROS levels and increased MDA levels (Fig. 2). MDA is one kind of the final products of phospholipid peroxidation responsible for cell membrane damage, and an increase in lipid peroxidation under stress always parallels an increased production of ROS^22^. Moreover, we also found that the expression of a *PLD* gene, which encodes phospholipid hydrolase involved in phospholipid production, was upregulated only in the infected RG leaves and not in the infected SY leaves (Fig. 2)^25^. Higher plants avoid oxidative damage by increasing their levels of endogenous antioxidant defenses^22^. Notably, antioxidant levels were elevated only in the infected SY leaves and not in the infected RG leaves (Fig. 2), which may be an important factor for resistance to *B. cinerea*.

### Transcriptional regulatory networks, including those involving *WRKY* and *MYB* genes, are associated with the ABA pathway

Induced expression of *MYB*s has been observed in normal ripe and in noble rotted (atypical gray mold disease) or bunch rotted (typical gray mold disease) grape berries in response to *B. cinerea*^17,18,20^ and has been suggested to be a common response in plants against *B. cinerea*^15,37^. Furthermore, knocking out *VvWRKY52* in *V. vinifera* Thompson Seedless was reported to increase resistance to *B. cinerea*^38^. In the current study, the expression of >20 *WRKY* genes and >20 *MYB* genes was significantly induced in both the infected RG leaves and the infected SY leaves (Supplemental Table 5); however, the specific roles of these genes in grape resistance to *B. cinerea* have yet to be characterized. We found that genes involved in the ABA pathway were associated with WRKY and MYB regulatory modules during *B. cinerea* infection (Fig. 3). It is known that negative regulation of ABA signaling by WRKY33 enhances the immunity of *A. thaliana* to *B. cinerea*^13^. Our data suggest that the ABA pathway was suppressed in the infected SY leaves but not in the infected RG leaves, since the expression of the ABA repressor *PP2C* genes^39^ was induced only in the infected SY leaves (Fig. 3). Thus, we hypothesize that the regulation of the ABA pathway through *WRKY* and *MYB* genes is a contributing factor in the resistance of grape leaves to *B. cinerea*.

### Differences in C/N metabolism may contribute to the contrasting resistance levels of Shuangyou and Red Globe

Compared with the SY leaves, the RG leaves exhibit a lower degree of downregulated expression of a series of genes associated with photosynthesis during infection, which may indicate an insufficient C supply in infected RG leaves (Fig. 4). Photosynthesis has been reported to be downregulated at 48 hpi in lettuce (*Lactuca sativa*) and *A. thaliana* leaves after *B. cinerea* infection, and this phenomenon is recognized as a typical plant response to pathogens^15,37^. Defense responses involving immune reactions and biosynthesis of protective compounds required a high demand of energy^37^. Here, *B. cinerea* indeed caused the expression of genes associated with EMP and the TCA cycle to be more strongly upregulated in the infected RG leaves than in the infected SY leaves, which is in accordance with a higher upregulated expression of C transport-related genes in the infected RG leaves (Fig. 4). Infection by *B. cinerea* was reported to trigger upregulation of *VvSWEET4* expression in *V. vinifera* leaves and *VvSWEET7* expression in *V. vinifera* berries; these two genes encode sugar uniporters^40^.

Genes associated with glutamate metabolism were differentially expressed in the two infected genotypes (Fig. 4). The *GAD* and *GDH* genes are involved in the GABA (γ-aminobutyrate) shunt, which connects glutamate metabolism to the TCA cycle and has been linked to plant defense against phytopathogens^26^. GAD is responsible for transforming Glu into GABA and producing succinate, a metabolic TCA cycle intermediate^26^. Overactivation of the GABA shunt for replenishment of the TCA cycle can help control the defense-associated HR and enhance resistance to *B. cinerea* in the tomato ABA-deficient *sitiens* mutant^41^. Considering the higher transcript levels of *GAD* in the infected RG leaves than in the infected SY leaves (Fig. 4), we suggest that there might also be a higher activation of the GABA shunt, which may represent a component of the defense response. In contrast, *GDH* is a senescence-associated gene and is responsible for generating Glu from a TCA intermediate to link the TCA and GS/GOGAT cycles^26^. GDH has been suggested to facilitate colonization by the necrotrophic fungus *Cochliobolus miyabeanus* of susceptible rice by depleting components of the TCA cycle through GDH-mediated export of Glu for central glutamate metabolism^26^. Compared with the infected SY leaves, the infected RG leaves showed higher expression of several *GDH* genes (Fig. 4), and we propose that this may reduce the availability of compounds for the TCA cycle, thereby increasing susceptibility. ODC has been suggested to be responsible for forming putrescine, the main precursor of other polyamines^26^, and grape leaves preexposed to osmotic stress and later inoculated with *B. cinerea* present polyamine accumulation and enhanced susceptibility to *B. cinerea*^42^. The elevated expression of the *ODC* gene in the RG leaves but not in the SY leaves after *B. cinerea* infection (Fig. 4) may also be a factor in the different resistance levels of the two genotypes.

We conclude that *B. cinerea* infection caused a greater disturbance in C/N metabolism and thus required a more continuous supply in the RG leaves than in the SY leaves to keep the TCA cycle and glutamate metabolism from being exhausted. Assimilative N must be constantly replenished to supply glutamate metabolism in a resistant host, because the compounds associated glutamate metabolism provide both C skeletons and α-amino groups^26^. High N concentrations in tomato reduce susceptibility to *B. cinerea*^43^, and our data also show that, compared with the infected RG leaves, the infected SY leaves exhibited higher expression of an *NR* gene and two *GS1* genes (Fig. 4), which might contribute to assimilation of N and to Glu synthesis, respectively^26^.

### Preinfection attributes of Shuangyou leaves contribute to the *B. cinerea* resistance

WGCNA revealed higher basal expression of a series of genes associated with carbohydrate, lipid, and Glu metabolism in control SY leaves than in control RG leaves (Fig. 5 and Supplemental Fig. 9). This suggests that SY leaves may have more carbon cycle- and energy-related intermediates than RG leaves do prior to inoculation with *B. cinerea*. Compared with the control RG leaves, the control SY leaves also presented higher *CAT* transcript levels and higher CAT activities (Fig. 2 and Supplemental Fig. 9). This suggests a higher basal antioxidative capacity in the SY leaves, which is unfavorable for *B. cinerea* infection^5,7^. Notably, the difference in the expression of both the *ANK* and *CPN* genes (Fig. 2 and Supplemental Fig. 9), which are associated with enhanced resistance to *B. cinerea* and negative regulation of cell death, respectively^44,45^, is consistent with the higher resistance of the SY leaves than the RG leaves against *B. cinerea*. This is important, since timely recognition of pathogens and effective signaling to activate immunity are crucial for disease resistance, and any delay can impair defense responses^46^.

### The *VaWRKY10* gene can enhance the resistance of grapevine against *B. cinerea*

Our data revealed resistance of SY berries and susceptibility of RG berries to *B. cinerea* (Fig. 6), as the resistance levels of the SY leaves and RG leaves contrasted. The expression of *WRKY10* in the berries was also differentially induced in response to *B. cinerea* infection in the two grapevine genotypes (Fig. 6). This sequence of this gene is highly similar to that of *AtWRKY18*, -*40* and -*60*, which are known to increase resistance to *B. cinerea*, correlating with the expression of the JA-regulated *PDF1.2* gene^29^. *VaWRKY10* from *V. amurensis* Shuangyou, which is involved in the defense response to *B. cinerea*, was then confirmed via a series of transgenic assays (Fig. 7). WRKY18 and WRKY40 have each been shown to bind to >1000 genetic loci, predominantly to W-box elements, and these proteins have been extensively studied for their function in basal defense against many pathogens^29,47^. Details regarding the regulation of *B. cinerea* resistance by *VaWRKY10* remain to be revealed, but this idea is interesting for future studies.

**Fig. 8:**
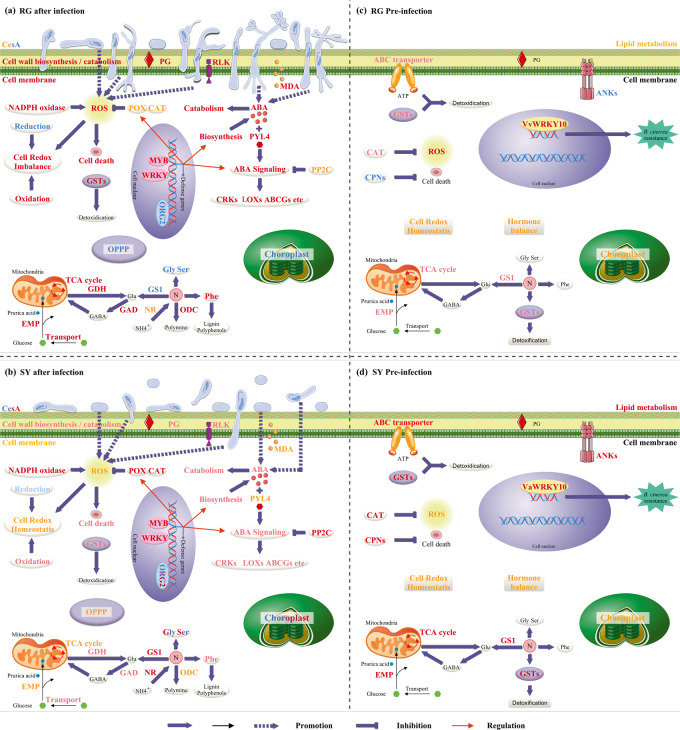
Putative defense mechanisms against *Botrytis cinerea* in susceptible RG and resistant SY leaves. **a** and **b** show the putative defense mechanisms of infected RG and SY leaves, respectively. The text in blue indicates genes whose expression was downregulated or the associated bioprocesses with sets of genes whose expression was downregulated. The text in red indicates upregulated expression, inscreased activities of CAT and POX, or increased levles of ABA and ROS. The text in light blue and pink shows slight down- and upregulated expression, respectively, and the text in yellow indicates no significant difference in expression, activities of CAT and POX and levels of ROS. **c** and **d** show basal expression differences between the control RG and SY leaves. The text in blue represents relatively low basal expression. The text in red indicates relatively high basal expression, CAT activities and ROS levels. Yellow indicates no significant difference. RG *Vitis vinifera* cv. Red Globe, SY *V. amurensis* Shuangyou

## Conclusions

This comparative transcriptomic study revealed a more substantial change in the transcriptome profile of RG leaves than in SY leaves during the early stages of infection by *B. cinerea (Fig.* *8**)*. Some of the putative defense mechanisms include the following: (1) early defense responses related to cell walls might facilitate the resistance of SY leaves to *B. cinerea*; (2) ROS accumulation and cell death processes facilitate the susceptibility of RG leaves to *B. cinerea*; (3) regulation of the ABA pathway and ROS-responsive genes through *WRKY* and *MYB* genes might play roles in grapevine resistance; (4) different responses in terms of C/N metabolism might contribute to the contrasting resistance levels; (5) preinfection attributes of SY leaves contribute to higher *B. cinerea* resistance because of higher basal expression of genes associated with C/N metabolism, antioxidant metabolism, and immunity to *B. cinerea* compared with those of RG leaves; and (6) high basal expression of *VaWRKY10* in grapevine should help in the defense against *B. cinerea*.

## Materials and methods

### Grape materials, *B. cinerea* disease assays, and physiological studies

RG, SY, and Thompson Seedless plants were maintained in a vineyard overseen by the grape germplasm and breeding program at Northwest A&F University, Shaanxi, China. Detached grape leaves and berry assays, statistical analysis and microexamination of fungi and lesion development as well as the measurement of CAT and POD activities were all carried out as previously described^7^. The detection of ABA levels was performed using a high-pressure liquid chromatography (HPLC) system at a testing center at China Agricultural University as previously described^48^. Levels of H_2_O_2_, MDA, proteins and chlorophyll were measured using previously published methods^49^.

### RNA extraction

Samples were collected from three biological replicates over time, and total RNA extraction was carried out using an E.Z.N.A. Plant RNA Kit (Omega Bio-tek, Norcross, GA, USA). The RNA quality and quantity were assessed on a 1.2% denatured agarose gel and a NanoDrop 1000 Spectrophotometer (Thermo Scientific, Wilmington, DE, USA), respectively.

### Illumina sequencing and data processing

Strand-specific grape RNA-seq libraries were constructed by Polar Genomics Company and sequenced via the single-end mode on an Illumina HiSeq 2000/2500 platform^50^. A total of 5–10 million reads with a length of 100 bp were generated for each library. Raw read and transcript filtering were performed as previously described^51^, and the final assembled transcripts were aligned to the 12× PN40024 genome and *B. cinerea* B05.10 genome assemblies^52,53^ using BLAT^54^, with a sequence identity ≥97%. Following alignment, the counts of mapped reads from each sample were derived and normalized to reads per kilobase of exon model per million mapped reads (RPKM). Genes that were differentially expressed between *B. cinerea-*inoculated samples and control samples were identified using the DESeq 1.8.3 package^55^. Genes with an adjusted *P* value ≤ 0.05 and at least a twofold change in expression were regarded as DEGs.

### Data analysis

The ‘Calculate and draw custom Venn diagrams’ system (http://bioinformatics.psb.ugent.be/webtools/Venn/) was used to construct Venn diagrams. Correlations and PCA between samples were performed using an online tool (http://www.omicshare.com/tools). Gene Ontology (GO) categories associated with the sets of DEGs were determined using the Plant MetGenMAP system^56^. Gene clustering was performed by K-means clustering using Genesis software^57^. TFs were confirmed online using Hmmscan alignment (http://www.plantTFdb/animalTFdb). TF-binding sites (TFBSs) in promoter sequences corresponding to the region 2 kb upstream of the transcription start site of genes were analyzed using the JASPAR database^58,59^. A hypergeometric test was used to generate *P* values adjusted using the Benjamini–Hochberg correction^24^. WGCNA was performed as previously described^27^.

### qRT-PCR and semiquantitative reverse-transcription PCR

Primers were designed using Primer 5 software;^60^ RNA extraction, DNase treatment and reverse transcription were carried out as previously described^61^. qRT-PCR analyses were carried out using SYBR Premix Ex Taq II (TaKaRa Biotechnology) on a Bio-Rad iQ5 thermocycler (Bio-Rad, Hercules, CA, USA). *VvActin1* (ID: XM_002282480.4; primer sequence, F: GATTCTGGTGATGGTGTGAGT, R: GACAATTTCCCGTTCAGCAGT) and *AtActin1* (ID: AT2G37620; primer sequence, F: GGCGATGAAGCTCAATCCAAACG, R: GGTCACGACCAGCAAGATCAAGACG) were used as reference genes. Relative log_2_ induction ratios of treated samples compared with those under the control treatment were calculated based on the DD_△t_ method^48^. Three biological replicates and three technical replicates were analyzed for each experiment. Semiquantitative reverse-transcription PCR was performed as previously described^28^, with each reaction repeated three times and the three independent analyses showing the same trends for each gene and sample.

### Transformation of *A. thaliana* and *V. vinifera* with *VaWRKY10*

*VaWRKY10* and *VvWRKY10* coding sequences were amplified as previously described^61^ using PrimeSTAR HS DNA Polymerase (TaKaRa Biotechnology) with gene-specific primers (F: 5′-CGGGATCCATGGAATTCGAATTTATTGATAC-3′, *BamH* I site underlined; R: 5′-TCCCCCGGGTCACCATTTTTCTATCTGAG-3′, *Sma* I site underlined). The sequences were analyzed using the BLAST program (http://Ncbi.nlm.Nih.gov/blast) of the NCBI database^61^. The *VaWRKY10* coding sequence was then fused to the CaMV 35S promoter in a pCAMBIA2300 vector^61^. The pCAMBIA2300-35S-*VaWRKY10* vector was then introduced into *A. thaliana* using the floral-dip method, and 75 mg/L kanamycin was used for identification of transgenic seedlings^62^. The *A. thaliana* plants were grown under 50–60% relative humidity at 21–23 °C and a long-day photoperiod (16 h light/8 h dark). The pCAMBIA2300-35S-*VaWRKY10* overexpression vector was then introduced into *A. tumefaciens* strain GV3101. *A. tumefaciens* cultures containing the plasmid were used to transform somatic embryos of the grape genotype Thompson Seedless (Fig. 6c); plantlet propagation was performed at 25 ± 1 °C and a 16 h photoperiod^38^.

### *A. thaliana* and Thompson Seedless *B. cinerea* resistance assays

Detached leaf assays^7^ were used to identify the resistance levels to *B. cinerea* of same-sized leaves of 4-week-old transgenic *A. thaliana* seedlings, two-month-old transgenic Thompson Seedless seedlings and their corresponding WT lines. Three biological replicates were analyzed. Droplets containing 1.5 × 10^6^/mL *B. cinerea* spores were applied to 15-18 *A. thaliana* leaves per replicate, and the infection was evaluated four days after inoculation by measuring the diameter of the area of spreading lesions^61^. *B. cinerea* spore suspensions containing 1.5 × 10^6^ spores /mL were sprayed onto 15–18 Thompson Seedless grape leaves per replicate. The infection was then evaluated by calculating the percentage of spreading lesions on each leaf^7^.

## Supplementary information

Supplementary legends

Supplemental Fig. 1: The development of *Botrytis* *cinerea* on grape leaves of contrasting resistance levels

Supplemental Fig. 2: Correlation of RNA-Seq data from RG and SY leaf samples

Supplemental Fig. 3: The number of differently expressed genes in RG and SY leaves at indicated hours post inoculation of *B. cinerea* based on RNA-Seq data

Supplemental Fig. 4: Expression clusters of all DEGs from SY and RG in their response to *B. cinerea*

Supplemental Fig. 5: Heatmap of GO terms selected from all enriched GO terms at the indicated time points

Supplemental Fig. 6: Chlorophyll and total protein levels

Supplemental Fig. 7: Cluster dendrogram of modules constructed by WGCNA analysis

Supplemental Fig. 8: Heatmap of correlations between expression modules found by WGCNA analysis

Supplemental Fig. 9: Heatmap of genes involved in biological processes in the selected modules of ‘coral2’, ‘plum1’, ‘darkgreen4’ and ‘lightsteelblue’, marked by the corresponding background colors of the panels

Supplemental Fig. 10: a Coding and b Amino acid sequence alignment of *VvWRKY10* from RG leaves and *VaWRKY10* from SY leaves

Supplemental Fig. 11: *VaWRKY10* expression profiles in transgenic plants

Supplemental Table 1: Microscopy statistics and mapping statistics of RNA-Seq data

Supplemental Table 2: Differently expressed genes (DEGs) induced by *Botrytis cinerea* in RG and SY leaves and expression clusters based on all DEGs

Supplemental Table 3: Enriched GO terms based on differently expressed genes and expression clusters

Supplemental Table 4: Biology processes and genes selected from all enriched GO terms

Supplemental Table 5: Transcription factor (*TF*) genes and predicted WRKY, bHLH, ERF, MYB and NAC targets

Supplemental Table 6: Prediction of *WRKY*, *MYB*, *ERF*, *NAC* and *bHLH* gene regulatory networks based on expression clusters

Supplemental Table 7: Prediction of *WRKY*, *MYB* and *ERF* regulatory modules associated with functional categories

Supplemental Table 8: Biology processes and genes selected from enriched GO terms in the selected modules
