# Supplementary material for: Comparative transcriptomic analysis highlights contrasting levels of resistance of Vitis vinifera and Vitis amurensis to Botrytis cinerea
Source: Hortic Res. 2021 May 1;8:103. doi: 10.1038/s41438-021-00537-8 (PMC8087793; doi:10.1038/s41438-021-00537-8)
Supplement: Supplementary file 1 — Supplementary legends [file 41438_2021_537_MOESM1_ESM.pdf]

Supplemental legends

Supplemental Fig. 1: Development of *Botrytis cinerea* on grape leaves of contrasting resistance levels. Development of *B. cinerea* on RG **a** and SY **b** at the indicated hours post-inoculation (hpi) with *B. cinerea*, imaged via scanning electron microscopy. The scale bars in **a** are 20, 10, 100, 100, 100, and 100 μm, and the higher magnification image at 12 hpi is shown at a scale of 10 μm. The scale bars in **b** are 100, 50, 20, 20, 100, and 50 μm, and the higher magnification image at 36 hpi is shown at a scale of 10 μm. RG: *Vitis vinifera* cv. Red Globe; SY: *V. amurensis* Shuangyou.

Supplemental Fig. 2: Correlations of RNA-Seq data from RG and SY leaf samples. a Heatmap of Spearman's correlations of the expression levels in all samples compared to those of each other, represented by colors ranging from blue (0.4) to red (1). **b** Verification of RNA-seq data by quantitative real-time (qRT)-PCR. Principal component analysis based on all expressed genes in the RG **c** and SY samples **d**. Distances within the plot (PCA) correspond to the biological coefficient of variation between each pair of samples. I: leaves inoculated with *B. cinerea*; C: control leaves inoculated with sterile water; rep1-3 indicates the three biological replicates; RG: *Vitis vinifera* cv. Red Globe; SY: *V. amurensis* Shuangyou.

Supplemental Fig. 3: Number of differentially expressed genes in RG and SY leaves at the indicated hours post-inoculation of *B. cinerea* based on RNA-Seq data. RG: *Vitis vinifera* cv. Red Globe; SY: *V. amurensis* Shuangyou.

Supplemental Fig. 4: Expression clusters of all DEGs from SY and RG in response to *B. cinerea*. The number of genes is listed for each cluster. The X-axis indicates hours post-inoculation: a-f indicates 4, 8, 12, 18, 24 and 36 hpi. The Y-axis indicates the $\log_2$-transformed ratios of the DEGs. The maximum/minimum value is set to ± 6. RG: *Vitis vinifera* cv. Red Globe; SY: *V. amurensis* Shuangyou.

Supplemental Fig. 5: Heatmap of GO terms selected from all enriched GO terms

at the indicated time points. The darker colors from red to blue indicate that the GO terms are more significantly enriched. The size of circles or triangles represents the number of DEGs associated with the indicated GO term. The triangles represent upregulation, and the circles represent downregulation. RG: *Vitis vinifera* cv. Red Globe; SY: *V. amurensis* Shuangyou.

Supplemental Fig. 6: Chlorophyll and total protein levels. a Chlorophyll levels and **b** total protein levels of SY and RG samples at the indicated time points. The data represent the means of three experiments. The error bars represent the standard deviations. The different lowercase letters represent significant differences at $P \leq 0.05$ (Duncan's test). RG: *Vitis vinifera* cv. Red Globe; SY: *V. amurensis* Shuangyou; I: leaves inoculated with *B. cinerea*; C: control, leaves inoculated with sterile water.

Supplemental Fig. 7: Cluster dendrogram of modules constructed by WGCNA.

Supplemental Fig. 8: Heatmap of correlations between expression modules found by WGCNA.

Supplemental Fig. 9: Heatmap of genes involved in biological processes in the selected modules of 'coral2', 'plum1', 'darkgreen4' and 'lightsteelblue', marked by the corresponding background colors of the panels. RG: *Vitis vinifera* cv. Red Globe; SY: *V. amurensis* Shuangyou; I: leaves inoculated with *B. cinerea*; C: control leaves inoculated with sterile water.

Supplemental Fig. 10: a Coding and b amino acid sequence alignment of the *VvWRKY10* gene from RG leaves and the *VaWRKY10* gene from SY leaves.

Supplemental Fig. 11: *VaWRKY10* expression profiles of transgenic plants. a Relative *VaWRKY10* expression levels in transgenic *Arabidopsis thaliana* leaves at the indicated time points after *Botrytis cinerea* infection. **b** Relative *VaWRKY10* expression levels in 11 transgenic two-month-old tissue-cultured Thompson Seedless seedlings and wild-type seedlings. The data represent the means ± standard deviations of three replicates, and 15 leaves were pooled for each replicate. The asterisks indicate significant differences between the treatment and mock samples (**, $P \leq 0.01$; *t*-test).

Supplemental Table 1: Microscopy statistics and mapping statistics of RNA-Seq data. A. Microscopy statistics of three biological replicates from *Botrytis cinerea*-inoculated RG and SY leaves. **B.** Mapping statistics. The read numbers and percentage of genes mapped to the *Vitis* genome and *B. cinerea* genome are given. RG: *Vitis vinifera* cv. Red Globe; SY: *V. amurensis* Shuangyou; I: leaves inoculated with *B. cinerea*; C: control leaves inoculated with sterile water; rep: replicate.

Supplemental Table 2: Differently expressed genes (DEGs) whose expression was induced by *Botrytis cinerea* in RG and SY leaves and expression clusters based on all DEGs. A. All DEGs filtered by ratios ≥ 2 or ≤ 0.5 and adjusted *P*-values ≤ 0.05 among the genes in the indicated samples. **B.** DEGs in the indicated expression cluster identified through the K-means method based on the pooling of all DEGs. The values are $\log_2$(ratios). RG: *Vitis vinifera* cv. Red Globe; SY: *V. amurensis* Shuangyou.

Supplemental Table 3: Enriched GO terms based on differently expressed genes and expression clusters. A. All enriched GO terms of the biological process Gene Ontology category with *P*-values ≤ 0.05, based on the pooling of all DEGs from RG and SY samples at each time point. **B.** All enriched GO terms based on genes in the indicated expression clusters. RG: *Vitis vinifera* cv. Red Globe; SY: *V. amurensis* Shuangyou.

Supplemental Table 4: Biological processes and genes selected from all enriched GO terms. A. Biology processes among enriched GO terms at each time point. The green color represents sets of genes whose expression is downregulated from the indicated GO terms, and the expression of the others is upregulated. **B.** Genes selected from the enriched GO terms associated with key biological processes. RG: *Vitis vinifera* cv. Red Globe; SY: *V. amurensis* Shuangyou.

Supplemental Table 5: Transcription factor (*TF*)-encoding genes and predicted WRKY, bHLH, ERF, MYB and NAC targets. A. All *TF*-encoding genes differentially expressed in RG and SY after inoculation with *Botrytis cinerea* and selected genes that whose expression was highly significantly induced. **B.** Genes that

contain WRKY-, bHLH-, ERF-, MYB- and NAC-binding motif(s) in their promoter regions. The motif position is given within the 2 kb upstream promoter sequence relative to the promoter start site (i.e., position 1,000 is directly before the transcription start site). RG: *Vitis vinifera* cv. Red Globe; SY: *V. amurensis* Shuangyou.

Supplemental Table 6: Prediction of *WRKY*, *MYB*, *ERF*, *NAC* and *bHLH* gene regulatory networks based on expression clusters. A. Numbers of *WRKY*, *MYB*, *ERF*, *NAC* and *bHLH* genes in the expression clusters. **B.** Hypergeometric test for enrichment of *WRKY*, *MYB*, *ERF*, *NAC* and *bHLH* genes in the 23 gene expression clusters. **C.** Numbers of genes that contain WRKY-, MYB-, ERF-, NAC- and bHLH-binding motifs within a 2 kb region upstream of the transcription start site in the 23 gene expression clusters. **D.** Hypergeometric test for enrichment of genes that contain WRKY-, MYB-, ERF-, NAC- and bHLH-binding motifs within a 2 kb region upstream of the transcription start site in the 23 gene expression clusters. Adjusted *P*-values ≤ 0.05 are highlighted in red.

Supplemental Table 7: Prediction of *WRKY*, *MYB* and *ERF* regulatory modules associated with functional categories. A. Genes from cluster 3 containing WRKY- and MYB-binding motif(s) in their promoter regions and their functional categories. **B.** Genes from cluster 5 containing WRKY-binding motif(s) in their promoter region and their functional categories. **C.** Genes from cluster 13 containing WRKY- and ERF-binding motif(s) in their promoter regions and their functional categories. The motif position is given within a 2 kb upstream promoter sequence relative to the promoter start site (i.e., position 1,000 is directly before the transcription start site).

Supplemental Table 8: Biological processes and genes selected from enriched GO terms in the selected modules. A. Biological processes associated with enriched GO terms (q values ≤ 0.05) based on genes in the 'coral2', 'plum1', 'darkgreen4' and 'lightsteelblue' modules. **B.** Genes involved in selected biological processes from the modules described above. **C.** Several non-DEG *WRKY* genes displayed high expression levels in all RG and SY samples, with no significant difference between

control samples and samples infected by *Botrytis cinerea*. For the RPKM values, the expression levels of these non-DEG *WRKY* genes in all samples were mostly higher than the levels of the differentially expressed *WRKY* genes (caused by *B. cinerea*) in each corresponding sample. DEG: Differently expressed gene; RG: *Vitis vinifera* cv. Red Globe; SY: *V. amurensis* Shuangyou; I: leaves inoculated with *B. cinerea*; C: control, leaves inoculated with sterile water.
