# Supplementary figures and images for: Comparative transcriptomic analysis highlights contrasting levels of resistance of Vitis vinifera and Vitis amurensis to Botrytis cinerea
Source: Hortic Res. 2021 May 1;8:103. doi: 10.1038/s41438-021-00537-8 (PMC8087793; doi:10.1038/s41438-021-00537-8)

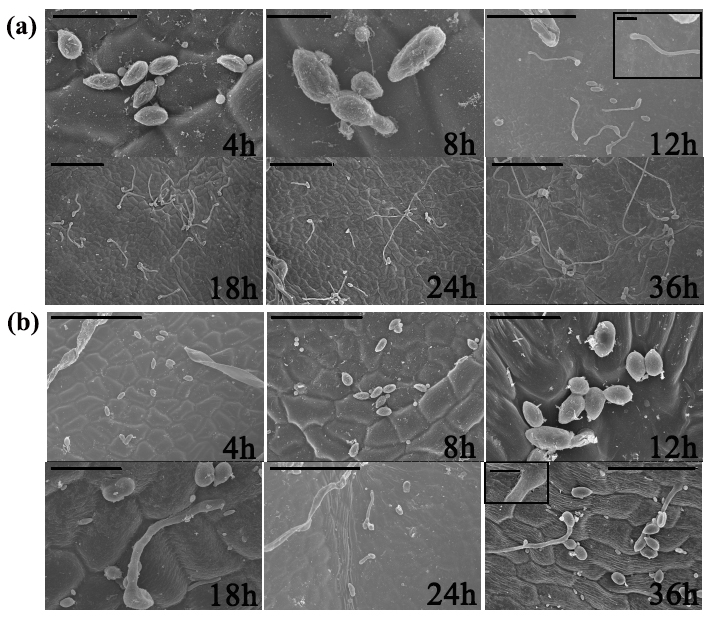

Supplement: Supplementary file 2 — Supplemental Fig. 1: The development of Botrytis cinerea on grape leaves of contrasting resistance levels [file 41438_2021_537_MOESM2_ESM.jpg]

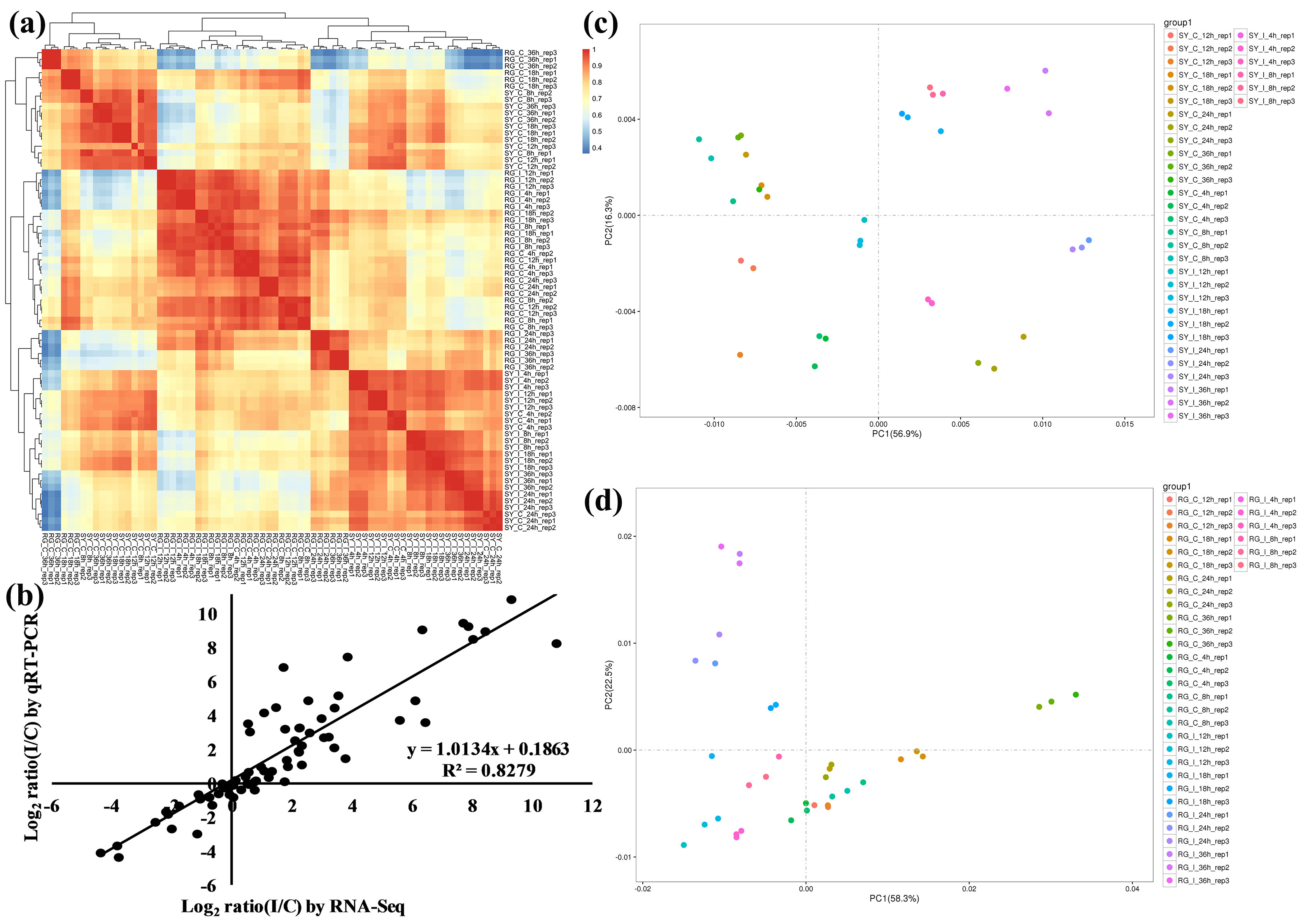

Supplement: Supplementary file 3 — Supplemental Fig. 2: Correlation of RNA-Seq data from RG and SY leaf samples [file 41438_2021_537_MOESM3_ESM.jpg]

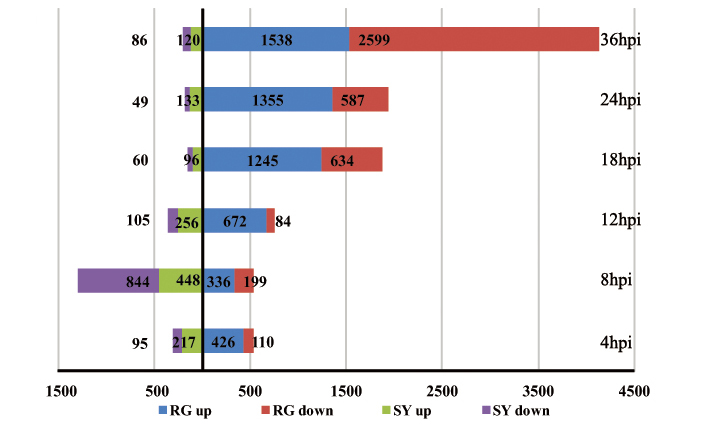

Supplement: Supplementary file 4 — Supplemental Fig. 3: The number of differently expressed genes in RG and SY leaves at indicated hours post inoculation of B. cinerea based on RNA-Seq data [file 41438_2021_537_MOESM4_ESM.jpg]

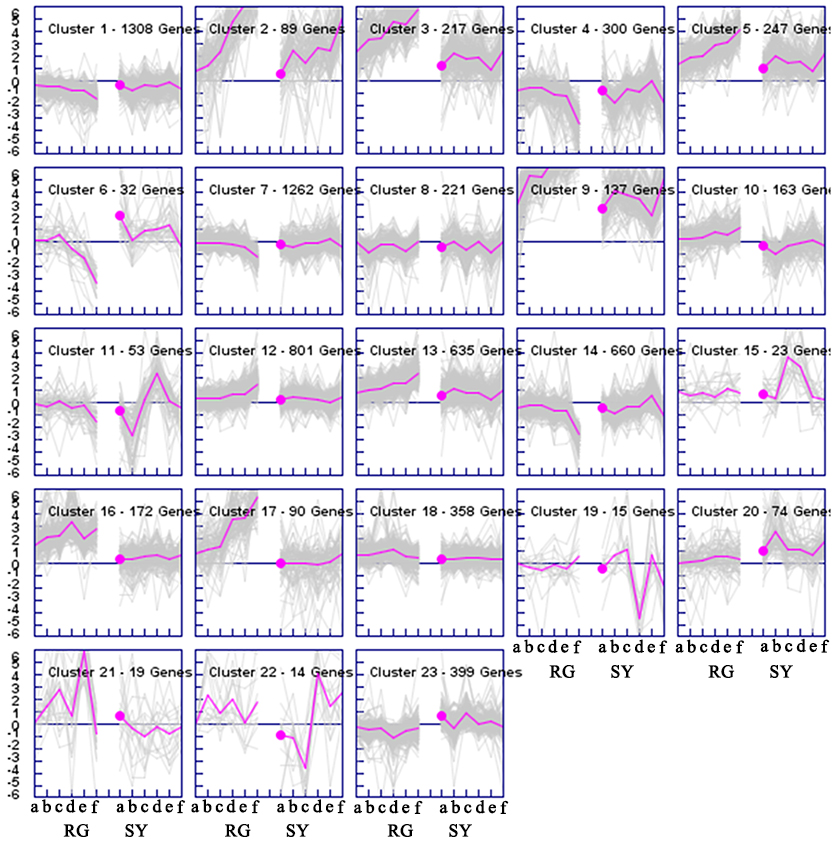

Supplement: Supplementary file 5 — Supplemental Fig. 4: Expression clusters of all DEGs from SY and RG in their response to B. cinerea [file 41438_2021_537_MOESM5_ESM.jpg]

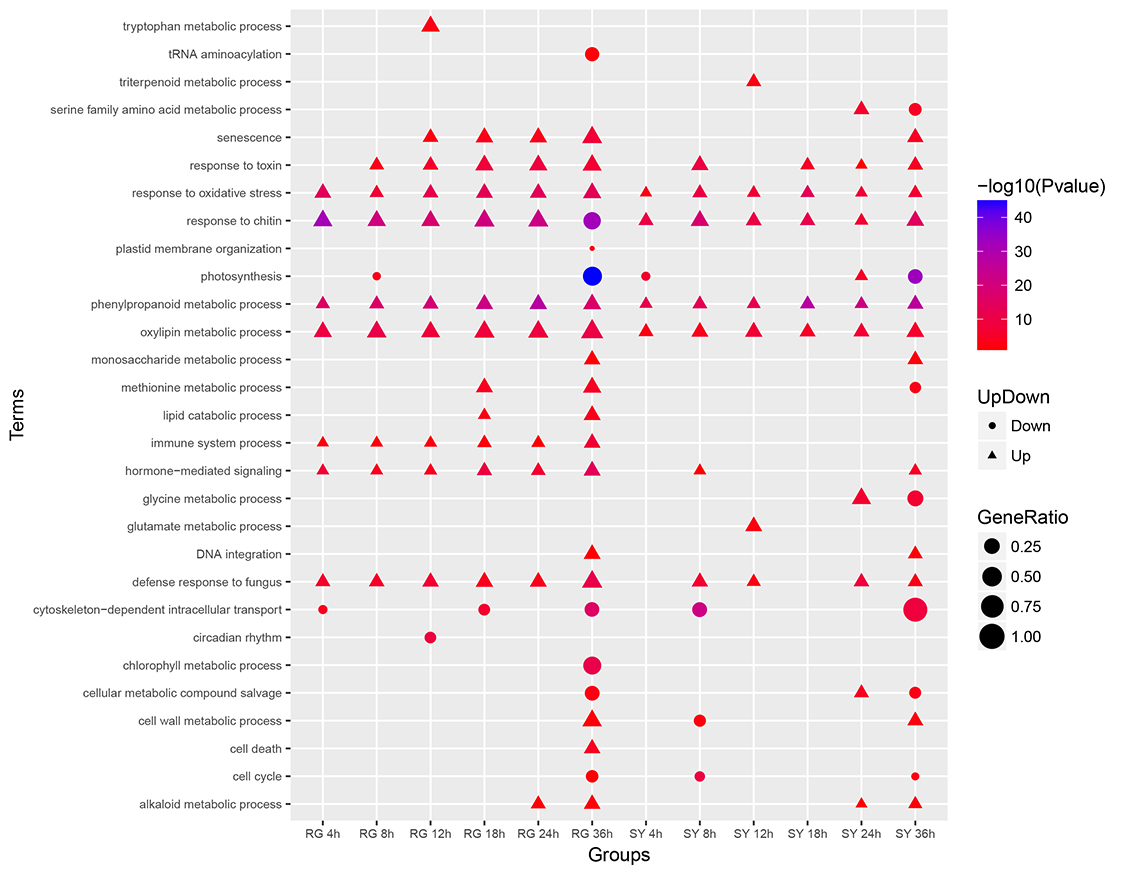

Supplement: Supplementary file 6 — Supplemental Fig. 5: Heatmap of GO terms selected from all enriched GO terms at the indicated time points [file 41438_2021_537_MOESM6_ESM.jpg]

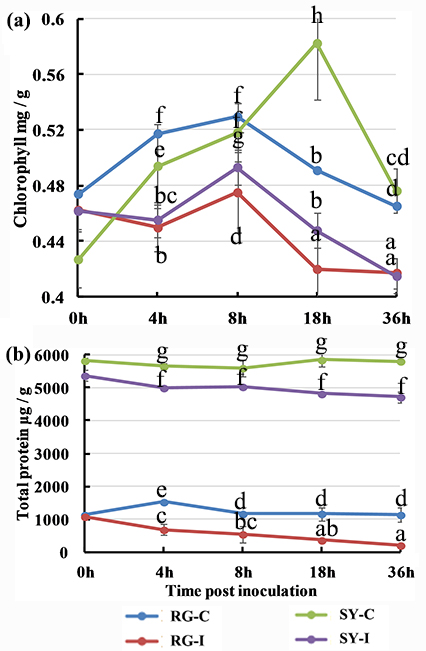

Supplement: Supplementary file 7 — Supplemental Fig. 6: Chlorophyll and total protein levels [file 41438_2021_537_MOESM7_ESM.jpg]

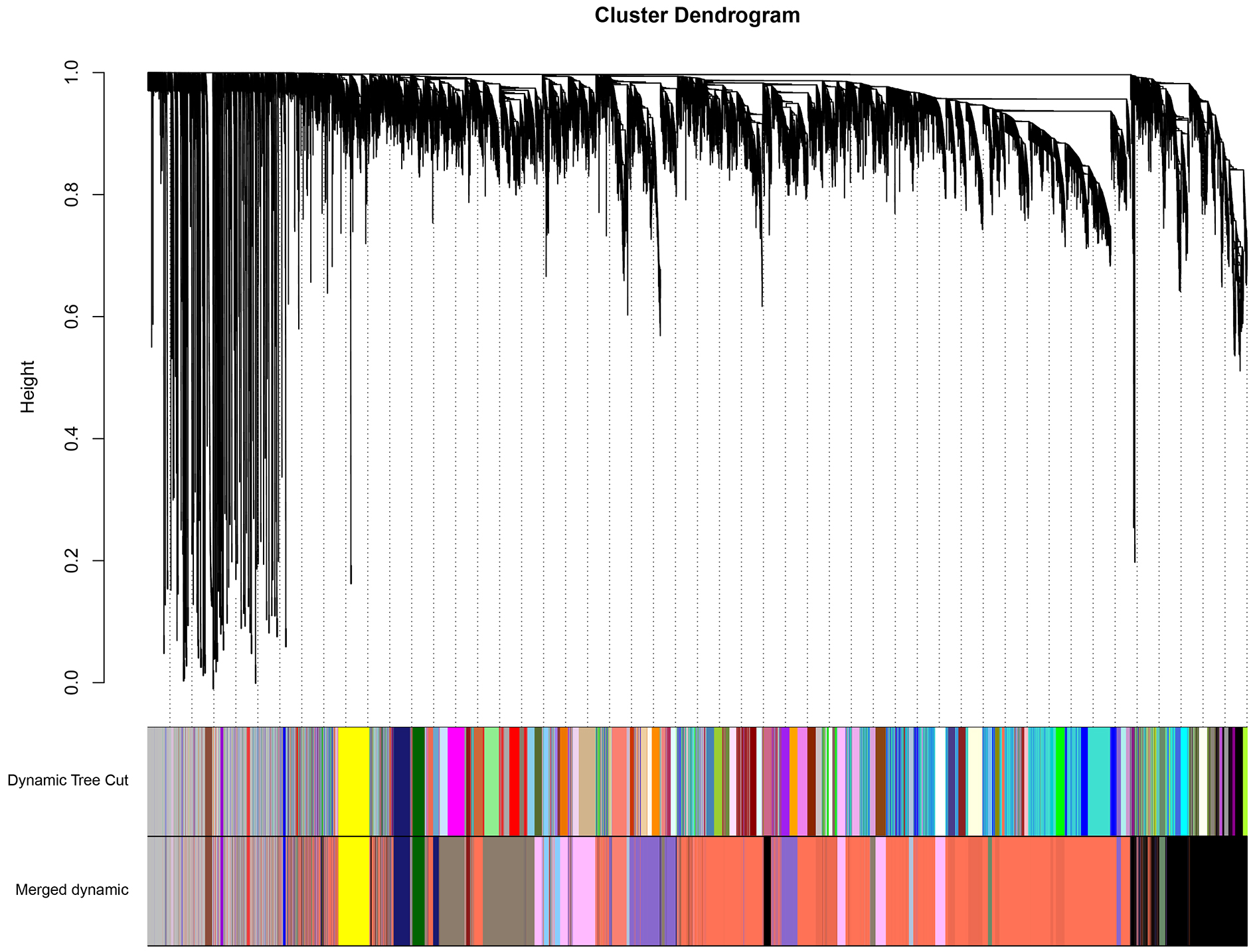

Supplement: Supplementary file 8 — Supplemental Fig. 7: Cluster dendrogram of modules constructed by WGCNA analysis [file 41438_2021_537_MOESM8_ESM.jpg]

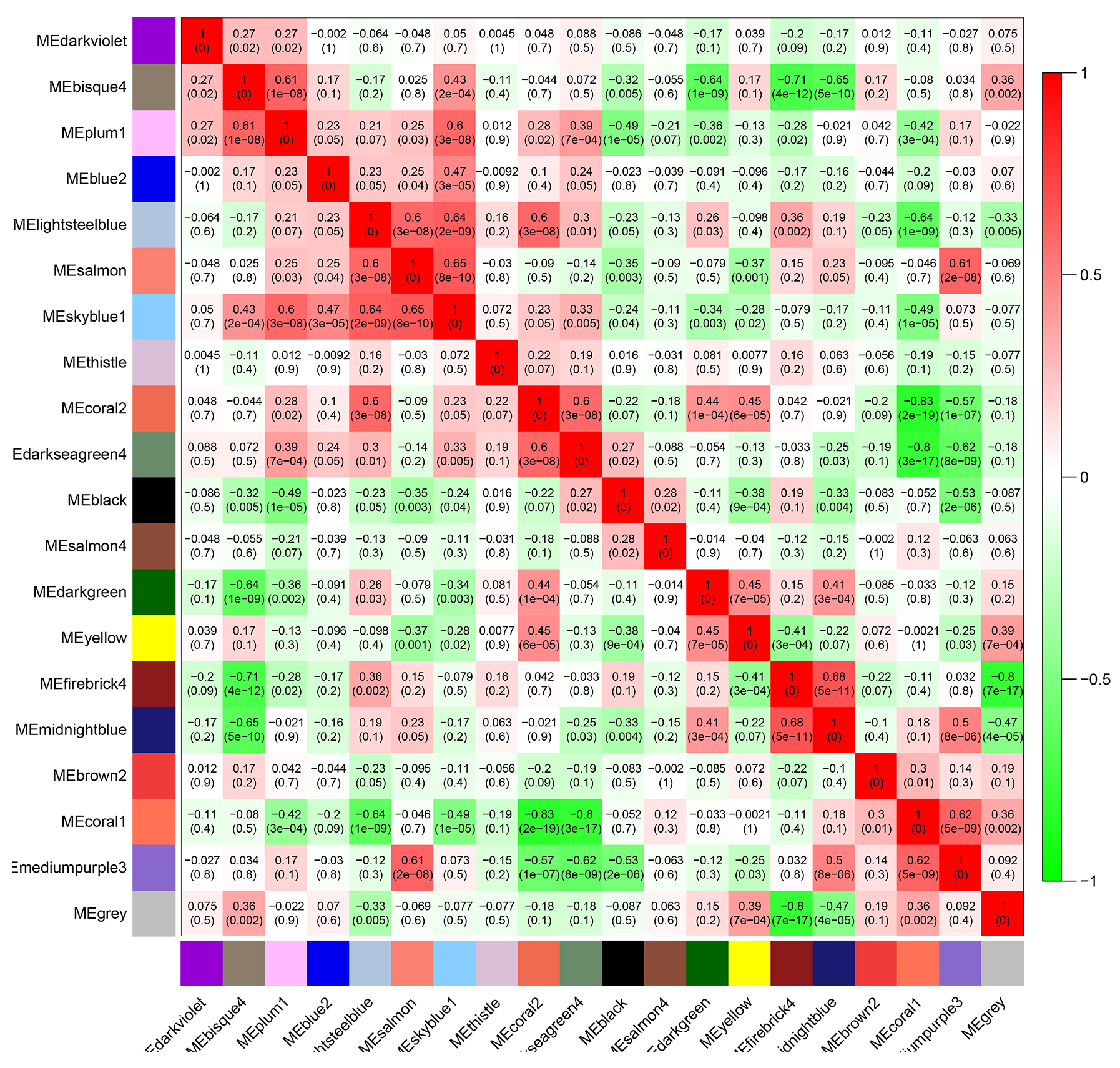

Supplement: Supplementary file 9 — Supplemental Fig. 8: Heatmap of correlations between expression modules found by WGCNA analysis [file 41438_2021_537_MOESM9_ESM.jpg]

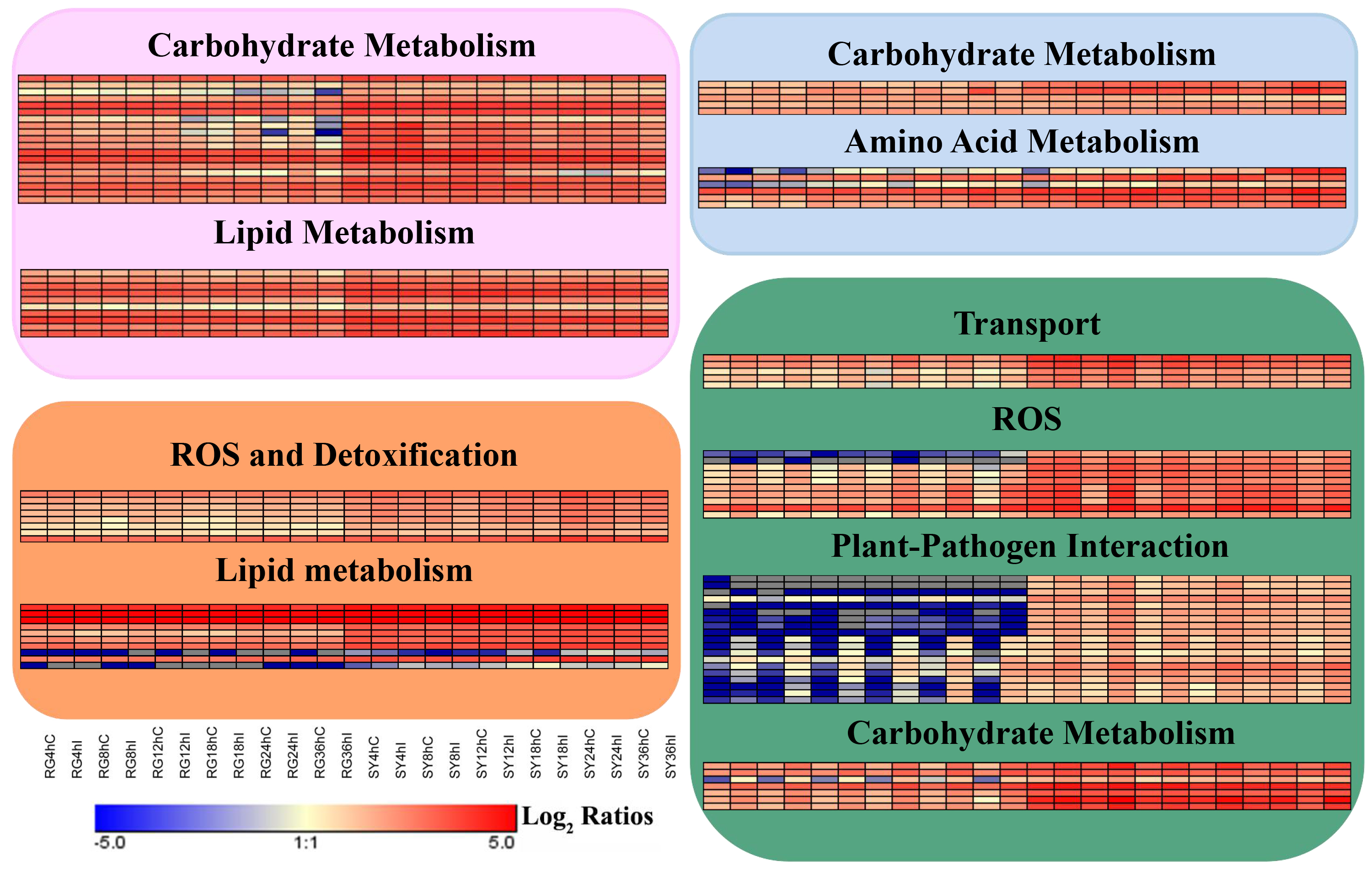

Supplement: Supplementary file 10 — Supplemental Fig. 9: Heatmap of genes involved in biological processes in the selected modules of ‘coral2’, ‘plum1’, ‘darkgreen4’ and ‘lightsteelblue’, marked by the corresponding background colors of the panels [file 41438_2021_537_MOESM10_ESM.jpg]

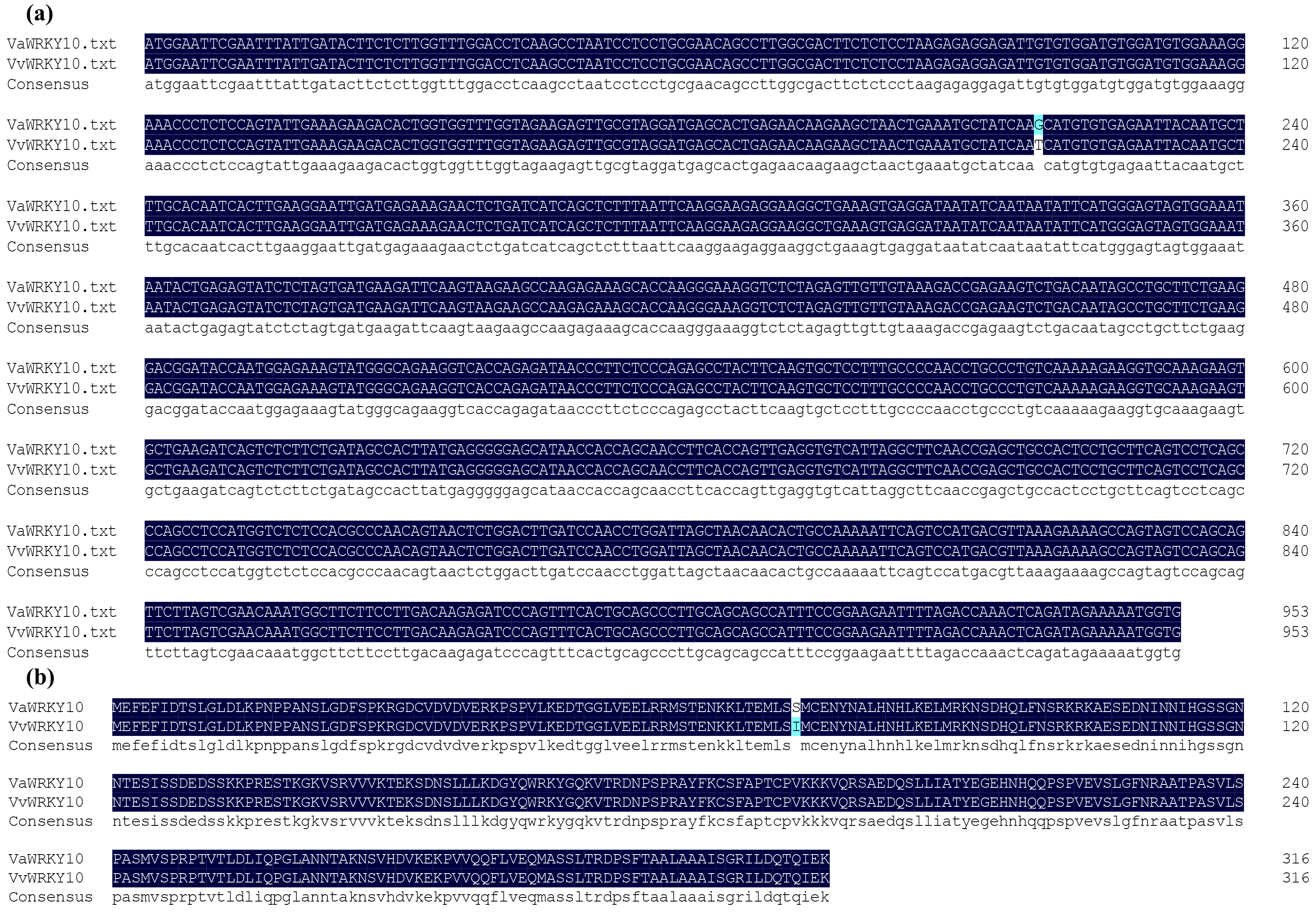

Supplement: Supplementary file 11 — Supplemental Fig. 10: a Coding and b Amino acid sequence alignment of VvWRKY10 from RG leaves and VaWRKY10 from SY leaves [file 41438_2021_537_MOESM11_ESM.jpg]

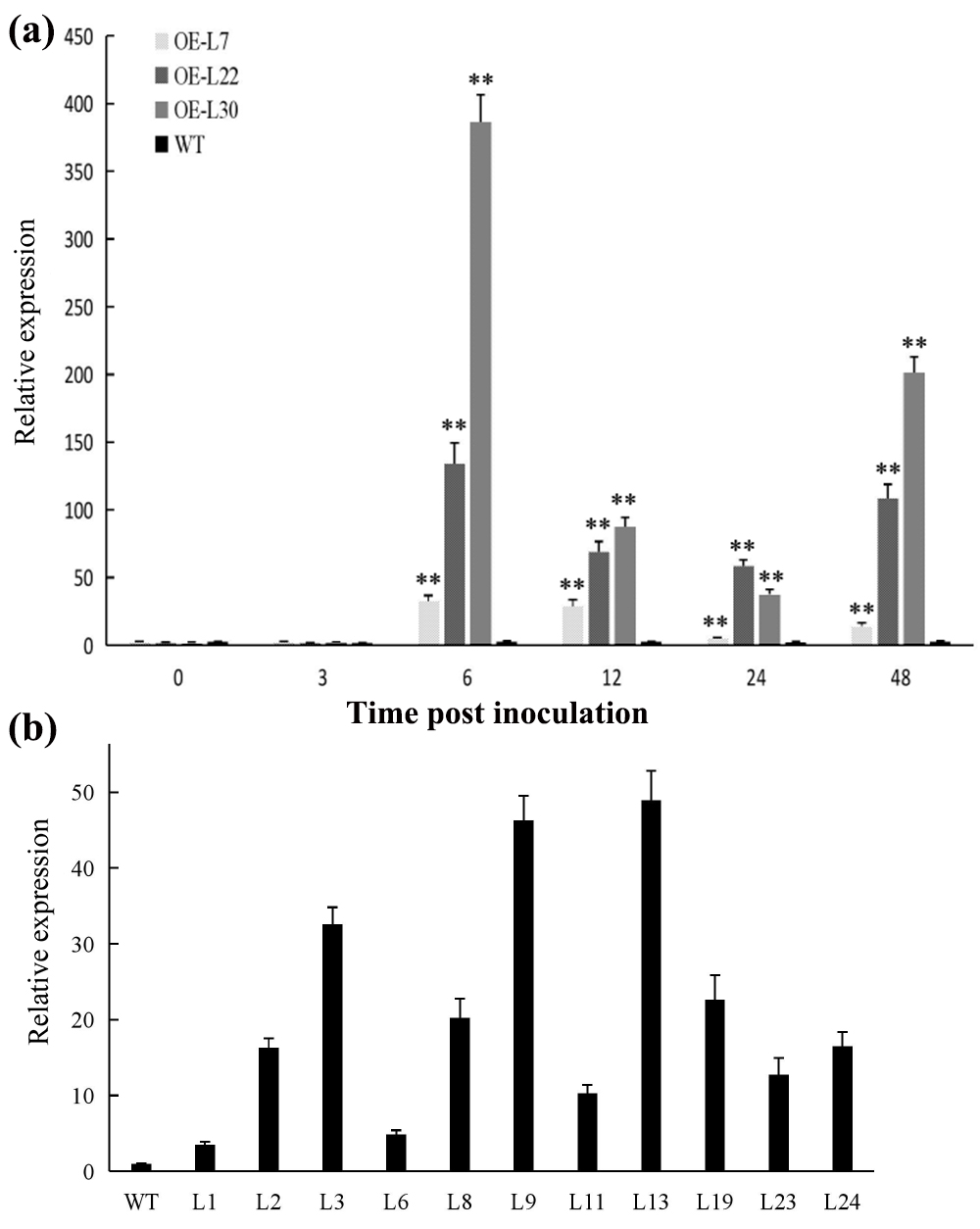

Supplement: Supplementary file 12 — Supplemental Fig. 11: VaWRKY10 expression profiles in transgenic plants [file 41438_2021_537_MOESM12_ESM.jpg]
